# Interpretable machine learning for prognostic prediction in critically ill patients with coronary artery disease: a multicenter study

**DOI:** 10.3389/fmed.2026.1794827

**Published:** 2026-03-30

**Authors:** Shu Yang, Shuo Zhang, Lianzheng Ma, Jinfang Zeng, Min Wang, Shunbin Huang, Xiao Zhang, Xiao Liang, Minmin Zhu

**Affiliations:** 1 Wuxi School of Medicine, Jiangnan University, Wuxi, China; 2 Department of Anesthesiology and Pain Medicine, Wuxi No. 2 People's Hospital (Jiangnan University Medical Center), Wuxi, China; 3 Tianjin Key Laboratory of Ionic-Molecular Function of Cardiovascular Disease, Department of Cardiology, Tianjin Institute of Cardiology, the Second Hospital of Tianjin Medical University, Tianjin, China

**Keywords:** coronary artery disease (CAD), critically ill, machine learning, MIMIC database, SHAP

## Abstract

**Background:**

Coronary artery disease (CAD) ranks among the most prevalent and clinically challenging cardiovascular disorders encountered in the intensive care unit (ICU). Patients with CAD admitted to the ICU typically exhibit elevated mortality rates, intricate pathophysiological alterations, and a high likelihood of adverse outcomes. This study aims to develop and validate a prognostic prediction model for ICU-admitted CAD patients using machine learning (ML) methodologies.

**Methods:**

The data were retrieved from two independent cohorts within the Medical Information Mart for Intensive Care (MIMIC) database: MIMIC-IV was utilized for model training, while MIMIC-III served as an external validation dataset. The primary endpoints of the prediction were the 28- and 365-day mortality risks in this patient population. Feature selection was performed using LASSO regression integrated with commonality analysis, and feature importance was quantified via the SHapley Additive exPlanations (SHAP) approach to identify critical risk factors. Subsequently, short-term and long-term mortality risk prediction models for patients with coronary artery disease were developed based on seven interpretable machine learning algorithms.

**Results:**

A total of 15,930 patients with coronary artery disease were enrolled in this study (mean age, 70.3 ± 12.1 years; 5,055 females, accounting for 31.7%). To evaluate the mortality risk of patients across different time horizons, we developed predictive models incorporating 40 and 41 feature variables, respectively. Comparative analyses with six other machine learning algorithms revealed that the RandomForest algorithm exhibited the optimal performance in predicting both short-term and long-term mortality risks among patients with coronary artery disease [28-day mortality risk: Internal validation: AUC = 0.858, 95% CI: 0.843–0.872; Accuracy = 88.2%; External validation: AUC = 0.914, 95% CI: 0.904–0.923; Accuracy = 91.4%] [365-day mortality risk: Internal validation: AUC = 0.851, 95% CI: 0.840–0.863; Accuracy = 79.6%; External validation: AUC = 90.1, 95% CI: 0.893–0.909; Accuracy = 85.3%].

**Conclusion:**

The random forest model developed in this study exhibited robust predictive performance and generalization capability in evaluating short-term and long-term mortality risks among critically ill patients with CAD. As a promising predictive tool, it offers data-driven decision support for clinicians to conduct early identification of high-risk patients and perform risk stratification, while its ultimate clinical utility remains to be further validated by prospective studies.

## 1 Introduction

Coronary artery disease (CAD) ranks among the leading contributors to global cardiovascular-related mortality and intensive care unit (ICU) admissions. Within the ICU setting, particularly for CAD patients complicated by acute myocardial infarction (AMI) or cardiogenic shock, mortality risk is markedly elevated. Existing evidence indicates that among AMI patients admitted to the ICU, the in-ICU mortality rate approximates 21.7%, with in-hospital mortality reaching 27.8% (1); for those requiring mechanical ventilation, in-hospital mortality further escalates to 42%−43% (2); and for patients with concurrent cardiogenic shock, in-hospital mortality may even exceed 40%−50% (3). These findings underscore the high complexity, rapid progression, and poor prognosis of critically ill CAD patients in the ICU, thereby emphasizing the critical clinical value of precise and timely risk assessment.

Currently, prognosis assessment for CAD patients in the ICU primarily relies on traditional clinical scoring systems or statistical models. While large-scale real-world databases offer rich resources for prognostic research, prediction tools based on conventional regression methods (e.g., Cox and logistic risk models) struggle to fully capture the complex clinical characteristics of patients (4, 5). Furthermore, existing studies have mostly focused on general critically ill populations, such as sepsis cohorts or mixed ICU populations, and lack targeted optimization for the unique pathological features of critically ill CAD patients (6). More importantly, many existing machine learning (ML) models lack interpretability (7), and most studies are based on a single database with insufficient external validation, which limits their clinical generalizability and reliability (8, 9).

With the continuous advancement of machine learning (ML) technology in the field of critical care medicine, accumulating evidence has demonstrated its notable advantages in handling high-dimensional nonlinear data features and enhancing the accuracy of prognostic prediction (10). However, such models suffer from a lack of interpretability, which restricts their widespread clinical application. Explainable machine learning approaches—such as SHapley Additive exPlanations (SHAP)—facilitate the elucidation of model decision-making mechanisms by quantifying and visualizing the magnitude of each variable's impact on prediction outcomes. This, in turn, enhances the transparency of the prediction process and improves the credibility and utility of these models in clinical decision support (11, 12).

This study aims to establish an efficient mortality risk prediction tool via machine learning, to support clinicians in individualized prognostic assessment and treatment decision optimization for CAD patients.

## 2 Materials and methods

### 2.1 Data source

This study conducted a retrospective cohort analysis using the MIMIC-IV and MIMIC-III databases, with MIMIC-III designated as the external validation cohort. The corresponding author, Yang Shu, has completed the Collaborative Institutional Training Initiative (CITI) program (Credential Number: 62274870), thereby obtaining full access privileges to the databases and being responsible for the extraction of clinical data related to CAD patients. The study was performed in strict compliance with the Strengthening the Reporting of Observational Studies in Epidemiology (STROBE) guidelines. Given that all personal identifiers in the MIMIC databases have been de-identified, this study was granted exemption from the requirement for obtaining written informed consent from patients.

### 2.2 Participants

**Figure 1:**
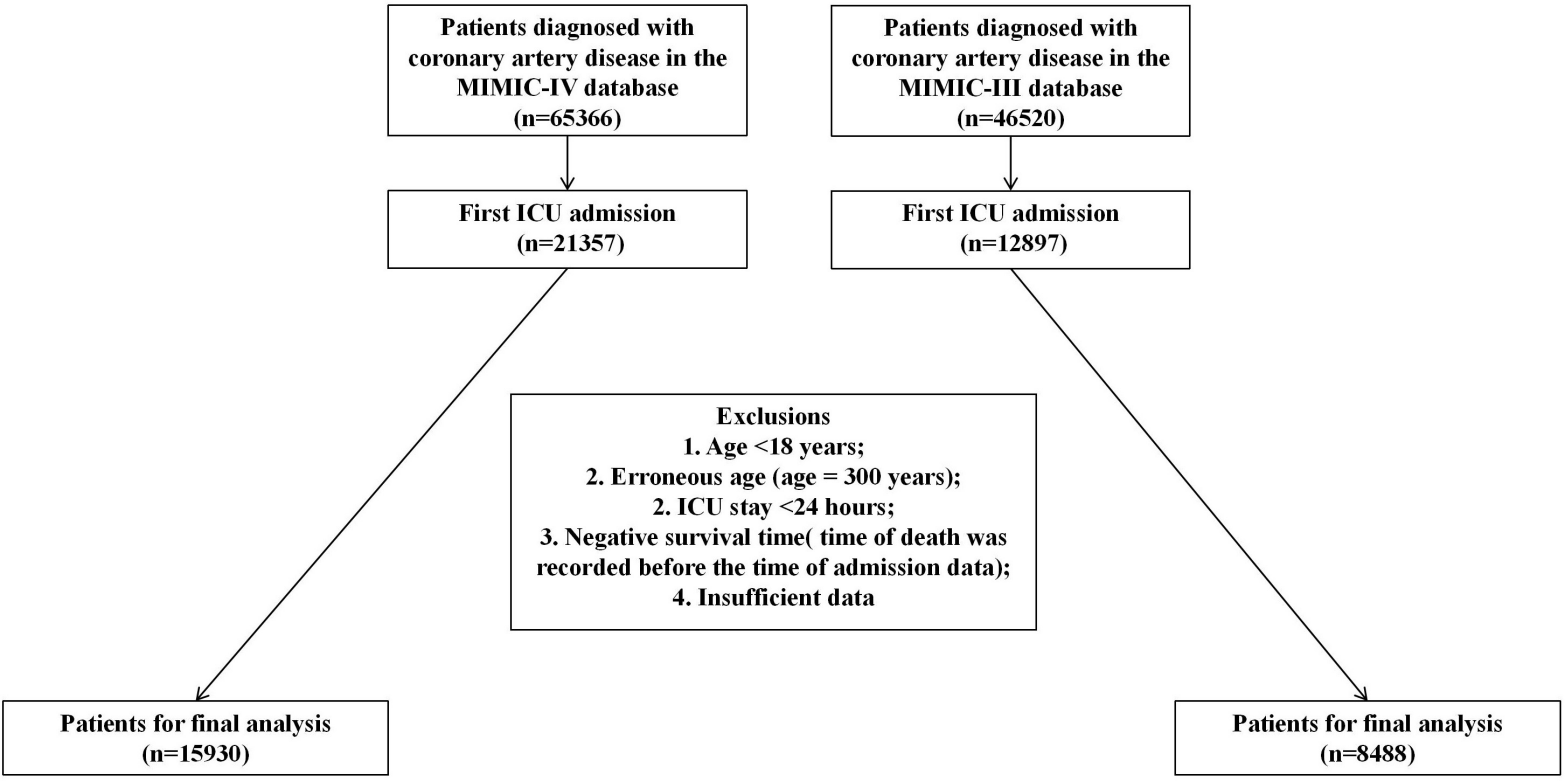
Participant screening process of critically ill coronary artery disease cohort study.

Considering that the present study aims to develop a universal initial risk screening tool for all critically ill patients admitted to the ICU with suspected or confirmed CAD, which is intended for application in real-world clinical scenarios where patients' diagnoses remain incompletely refined at the time of admission. This research identified patients with CAD using the International Classification of Diseases (ICD-9 and ICD-10) coding criteria, with ICD-9 codes 410–414 and ICD-10 codes I20–I22 and I25 (13). These codes encompass acute myocardial infarction, other acute ischemic heart diseases, and chronic ischemic heart diseases. This coding strategy has been adopted in prior MIMIC-based cardiovascular studies and is applicable to the investigation of typical CAD cases in epidemiological research. The full list of codes is provided in Supplementary Table 1. Eligible patients met the following inclusion criteria: (1) aged ≥ 18 years; (2) first admission to the ICU for treatment; (3) ICU length of stay (LOS) > 24 h. The detailed patient selection workflow is illustrated in Figure 1.

### 2.3 Outcome

The primary endpoints of this study were 28- and 365-day all-cause mortality following ICU admission, which were used to assess short-term and long-term survival outcomes in the cohort. Mortality data from both the MIMIC-IV and MIMIC-III databases were obtained by linking to the Master Death File (MDF) of the U.S. Social Security Administration (SSA). This linkage captures both in-hospital and post-discharge mortality events, thereby providing relatively comprehensive outcome data for follow-up analyses.

### 2.4 Data extraction

Relevant clinical data were extracted using Structured Query Language (SQL) from the PostgreSQL database. Demographic information was retrieved from the patients and admissions tables. ICU-related variables were extracted from the icustays table. Vital signs and laboratory test results were obtained from the chartevents and labevents tables, respectively. Comorbidities were identified from the diagnoses_icd table using predefined ICD codes. All predictor variables were restricted to values recorded within the first 24 h of ICU admission. For variables with multiple measurements within the 24-h window, selections were made based on relevant literature and clinical significance (e.g., mean values for laboratory tests or worst values for vital signs, as appropriate). Identical extraction logic and variable definitions were applied to both MIMIC-IV (derivation cohort) and MIMIC-III (external validation cohort) to ensure methodological consistency. These variables were pre-categorized into five clinically meaningful domains, informed by cardiovascular prognosis literature and decision-making frameworks in clinical practice: (1) Demographic characteristics: Sex, age, insurance type, and ethnicity; (2) Vital signs: Heart rate, SBP, DBP, MAP, respiratory rate, temperature, and SpO2; (3) Comorbidities: Myocardial infarction, congestive heart failure, peripheral vascular disease, dementia, cerebrovascular disease, chronic pulmonary disease, rheumatic diseases, peptic ulcer disease, liver disease, diabetes mellitus, paraplegia, renal disease, malignant cancer, solid tumors, and AIDS; (4) Laboratory parameters: Glucose, hematocrit, hemoglobin level, platelet count, WBC, blood urea nitrogen (BUN), creatinine, anion gap, serum sodium, potassium, calcium, chloride, bicarbonate, as well as coagulation parameters including INR, PT, and PTT; (5) Treatment interventions: Administration of aspirin, warfarin, statins, and vasoactive agents; receipt of CRRT and mechanical ventilation; additionally, hospitalization-related metrics such as total hospital length of stay (LOS) and ICU LOS were collected. The aforementioned variables are designed to capture the multidimensional determinants of prognosis in critically ill patients with coronary artery disease, thereby enhancing the interpretability of the model.

### 2.5 Statistical analysis

#### 2.5.1 Baseline data analysis

The normality of continuous variables was assessed using the Kolmogorov-Smirnov test. Normally distributed continuous variables were summarized as mean ± standard deviation (SD) and compared between groups using the independent-samples *t*-test. Non-normally distributed continuous variables were presented as median [first quartile (Q1), third quartile (Q3)] and analyzed for intergroup differences using the Wilcoxon rank-sum test. Categorical variables were reported as percentages (%), with intergroup comparisons performed using the chi-square test or Fisher's exact test. In cases of missing variables, those with a missing rate exceeding 30% shall be excluded, while variables with a missing rate below 30% shall undergo KNN imputation to mitigate potential biases in the model.

#### 2.5.2 Model development

During the model development phase, to construct a robust predictive model, candidate variables were first screened using LASSO regression combined with collinearity analysis. First, Lasso regression was applied to the training set. Leveraging L1 regularization, coefficients of variables with low contribution were shrunk to zero, thereby automating feature selection. The optimal regularization strength parameter (λ) was determined via 10-fold cross-validation, and the λ value under the “one-standard-error rule” (λ 0.1 se) was selected—this approach ensures model performance while yielding a more parsimonious and stable feature subset. Concurrently, the variance inflation factor (VIF) was used to diagnose multicollinearity among variables retained for model inclusion: variables with significant multicollinearity were either excluded or merged to ensure the stability and interpretability of the final model. The identical feature subset was employed to train all seven comparative machine learning algorithms, thereby ensuring the fairness of model comparisons.

Given that this study focuses on predicting the absolute risk of death at a fixed time point, classification models—unlike survival models, which output hazard ratios or risk functions—can directly generate the probability of death at a specific time point. This probability output is more amenable to clinical interpretation, enables direct comparison with decision thresholds, and facilitates decision curve analysis. Thus, machine learning algorithms were selected for this study. Seven machine learning predictive models were constructed using the filtered features, namely: XGBoost (Extreme Gradient Boosting), LR (Logistic Regression), LightGBM (Light Gradient Boosting Machine), RF (Random Forest), AdaBoost (Adaptive Boosting), SVM (Support Vector Machine), and KNN (K-Nearest Neighbors Algorithm). During model training, grid search combined with stratified 10-fold cross-validation was used for hyperparameter tuning to identify the optimal parameter configuration that maximized performance on the training set. The area under the curve (AUC) integrates sensitivity and specificity while remaining insensitive to classification thresholds. As a global standard metric for assessing a model's ranking performance—specifically its ability to distinguish between high-risk and low-risk patients—it is widely adopted and cross-referenced in comparable studies. Thus, model selection was based on the maximum area under the receiver operating characteristic curve (AUC-ROC)—the model with the highest AUC-ROC was designated as the optimal model, followed by internal and external validation. The overall predictive performance of the models was comprehensively assessed using multiple metrics, including AUC-ROC, accuracy, sensitivity, specificity, recall, and F1-score. The clinical utility of the final model was comprehensively evaluated across three dimensions: (1) Discrimination: The model's ability to distinguish outcome events was assessed using the AUC-ROC (as the primary metric), accuracy, and F1 score; (2) Calibration: The agreement between predicted probabilities and actual observed risks was visualized via a reliability curve (calibration plot), with the overall calibration error (CE) calculated for quantitative assessment; (3) Clinical decision utility: The clinical net benefit of the model across different decision thresholds was evaluated using decision curve analysis (DCA).

## 3 Results

### 3.1 Baseline characteristics

A total of 15,930 patients from the MIMIC-IV database were enrolled in this study, of whom 2,079 (≈13.0%) died within 28 days of ICU admission, and 4,063 (≈25.5%) died within 365 days. Additionally, an independent cohort of 8,488 patients was derived from a MIMIC-III subgroup that adhered to the identical inclusion criteria.

As presented in Table 1, the study population had a mean age of 70.3 ± 12.1 years, with 68.3% being male. Advanced age and White race were significantly associated with elevated short-term (28-day) and long-term (365-day) mortality risks. In contrast to total hospital length of stay, a longer ICU length of stay was associated with a stepwise increase in both short-term and long-term mortality risks. Among patients who died within 28 days, compared with survivors, they exhibited higher heart rate, respiratory rate, blood glucose levels, platelet count, WBC, anion gap, BUN, and creatinine levels at ICU admission—alongside more severe coagulation dysfunction. Additionally, on the first day of ICU stay, these patients had a higher rate of receiving interventions including warfarin, vasoactive agents, CRRT, and mechanical ventilation. For patients who died within 365 days, relative to survivors, they had lower systolic blood pressure, oxygen saturation, and serum chloride concentrations. They also had a significantly higher prevalence of multiple comorbidities, including congestive heart failure, peripheral vascular disease, dementia, cerebrovascular disease, chronic pulmonary disease, rheumatic diseases, peptic ulcer disease, liver disease, diabetes, paraplegia, malignant cancer, and solid tumors.

**Table 1:** Baseline characteristics comparison between the survival group and the non-survival group.

Variables	Total (*n* = 15,930)	28-day mortality		*P*-value	365-day mortality		*P*-value
		**Survivors (*****n*** = **13,851)**	**Non-survivors (*****n*** = **2,079)**		**Survivors (*****n*** = **11,867)**	**Non-survivors (*****n*** = **4,063)**	
Demographic							
**Gender**, ***n*** **(%)**				< 0.001			< 0.001
Female	5,055 (31.7)	4,272 (30.8)	783 (37.7)		3,531 (29.8)	1,524 (37.5)	
Male	10,875 (68.3)	9,579 (69.2)	1,296 (62.3)		8,336 (70.2)	2,539 (62.5)	
Age	70.3 ± 12.1	69.5 ± 12.0	75.4 ± 11.5	< 0.001	68.6 ± 11.8	75.1 ± 11.5	< 0.001
**Insurance**, ***n*** **(%)**				< 0.001			< 0.001
Medicare	10,652 (66.9)	8,990 (64.9)	1,662 (79.9)		7,384 (62.2)	3,268 (80.4)	
Medicaid	1,361 (8.5)	1,217 (8.8)	144 (6.9)		1,080 (9.1)	281 (6.9)	
Private	3,519 (22.1)	3,297 (23.8)	222 (10.7)		3,080 (26)	439 (10.8)	
Other	398 (2.5)	347 (2.5)	51 (2.5)		323 (2.7)	75 (1.8)	
**Race**, ***n*** **(%)**				< 0.001			0.049
White	10,982 (68.9)	9,637 (69.6)	1,345 (64.7)		8,192 (69)	2,790 (68.7)	
Black	980 (6.2)	849 (6.1)	131 (6.3)		694 (5.8)	286 (7)	
Asian	345 (2.2)	300 (2.2)	45 (2.2)		259 (2.2)	86 (2.1)	
Other	3,623 (22.7)	3,065 (22.1)	558 (26.8)		2,722 (22.9)	901 (22.2)	
Vital signs							
Heart rate (beats/min)	82.0 ± 13.9	81.3 ± 13.2	86.4 ± 17.1	< 0.001	81.2 ± 13.0	84.4 ± 16.2	< 0.001
SBP (mmHg)	116.1 ± 14.6	116.5 ± 14.2	113.6 ± 16.8	< 0.001	116.4 ± 13.8	115.3 ± 16.6	< 0.001
DBP (mmHg)	60.6 ± 10.0	60.6 ± 9.9	60.5 ± 10.6	0.489	60.6 ± 9.8	60.6 ± 10.6	0.978
MAP (mmHg)	76.8 ± 9.5	77.0 ± 9.4	75.6 ± 10.4	< 0.001	77.0 ± 9.2	76.1 ± 10.3	< 0.001
Respiratory rate (time/min)	19.0 ± 3.4	18.7 ± 3.2	21.0 ± 4.2	< 0.001	18.5 ± 3.1	20.3 ± 4.0	< 0.001
Temperature (°C)	36.8 ± 0.5	36.8 ± 0.4	36.7 ± 0.7	< 0.001	36.8 ± 0.4	36.7 ± 0.6	< 0.001
Spo2 (%)	97.0 ± 1.9	97.1 ± 1.7	96.5 ± 2.6	< 0.001	97.2 ± 1.7	96.7 ± 2.3	< 0.001
Laboratory parameters							
Glucose (mg/dl)	167.6 ± 99.3	162.1 ± 95.2	204.1 ± 117.0	< 0.001	158.8 ± 92.1	193.2 ± 114.2	< 0.001
Hematocrit (%)	30.0 ± 6.3	30.0 ± 6.3	30.1 ± 6.8	0.222	30.0 ± 6.2	30.1 ± 6.7	0.4
Hemoglobin (mg/dl)	9.9 ± 2.1	10.0 ± 2.1	9.8 ± 2.3	< 0.001	10.0 ± 2.1	9.8 ± 2.2	< 0.001
PLT (10^9^/L)	174.1 ± 84.6	172.7 ± 80.8	183.6 ± 106.2	< 0.001	170.0 ± 77.5	186.2 ± 101.7	< 0.001
WBC (10^9^/L)	13.5 (10.0, 17.9)	13.4 (10.0, 17.7)	14.1 (10.1, 19.5)	< 0.001	13.5 (10.2, 17.8)	13.3 (9.3, 18.4)	0.033
Anion gap (mmol/L)	12.2 ± 3.5	11.8 ± 3.3	14.4 ± 4.0	< 0.001	11.6 ± 3.2	13.9 ± 3.8	< 0.001
Bicarbonate (mmol/L)	21.6 ± 4.2	21.9 ± 3.9	19.8 ± 5.6	< 0.001	21.9 ± 3.7	20.7 ± 5.4	< 0.001
BUN (mg/dl)	22.0 (16.0, 35.0)	20.0 (15.0, 31.0)	37.0 (24.0, 58.0)	< 0.001	19.0 (15.0, 28.0)	34.0 (23.0, 54.0)	< 0.001
Chloride (mmol/L)	102.2 ± 5.7	102.5 ± 5.5	100.5 ± 6.9	< 0.001	102.8 ± 5.2	100.5 ± 6.7	< 0.001
Creatinine (mg/dl)	1.1 (0.9, 1.6)	1.1 (0.8, 1.5)	1.6 (1.1, 2.7)	< 0.001	1.0 (0.8, 1.4)	1.5 (1.0, 2.5)	< 0.001
Sodium (mmol/L)	139.4 ± 4.3	139.3 ± 4.0	140.0 ± 5.9	< 0.001	139.2 ± 3.8	139.7 ± 5.5	< 0.001
Potassium (mmol/L)	4.0 ± 0.5	4.0 ± 0.5	4.0 ± 0.7	0.51	4.0 ± 0.5	4.0 ± 0.6	0.101
INR	1.3 (1.2, 1.6)	1.3 (1.2, 1.5)	1.4 (1.2, 2.0)	< 0.001	1.3 (1.2, 1.5)	1.4 (1.2, 1.8)	< 0.001
PT (s)	17.2 ± 10.6	16.6 ± 9.3	21.1 ± 16.1	< 0.001	16.3 ± 8.5	19.7 ± 14.9	< 0.001
PTT (s)	33.7 (28.9, 50.5)	33.3 (28.8, 47.7)	37.8 (29.9, 70.0)	< 0.001	33.1 (28.8, 47.0)	35.9 (29.4, 61.2)	< 0.001
Comorbidities							
Hypertension (*n*, %)	6,117 (38.4)	5,560 (40.1)	557 (26.8)	< 0.001	5,023 (42.3)	1,094 (26.9)	< 0.001
Congestive heart failure (*n*, %)	6,598 (41.4)	5,389 (38.9)	1,209 (58.2)	< 0.001	4,160 (35.1)	2,438 (60)	< 0.001
Peripheral vascular disease (*n*, %)	2,604 (16.3)	2,189 (15.8)	415 (20)	< 0.001	1,760 (14.8)	844 (20.8)	< 0.001
Chronic pulmonary disease (*n*, %)	4,119 (25.9)	3,459 (25)	660 (31.7)	< 0.001	2,790 (23.5)	1,329 (32.7)	< 0.001
Rheumatic disease (*n*, %)	558 (3.5)	457 (3.3)	101 (4.9)	< 0.001	379 (3.2)	179 (4.4)	< 0.001
Peptic ulcer disease (*n*, %)	411 (2.6)	333 (2.4)	78 (3.8)	< 0.001	259 (2.2)	152 (3.7)	< 0.001
Liver disease (*n*, %)	1,117 (7.0)	812 (5.9)	305 (14.7)	< 0.001	621 (5.2)	496 (12.2)	< 0.001
Diabetes (*n*, %)	6,532 (41.0)	5,691 (41.1)	841 (40.5)	0.583	4,793 (40.4)	1,739 (42.8)	0.007
Paraplegia (*n*, %)	675 (4.2)	492 (3.6)	183 (8.8)	< 0.001	363 (3.1)	312 (7.7)	< 0.001
Renal disease (*n*, %)	4,405 (27.7)	3,579 (25.8)	826 (39.7)	< 0.001	2,730 (23)	1,675 (41.2)	< 0.001
Malignant cancer (*n*, %)	1,380 (8.7)	1,010 (7.3)	370 (17.8)	< 0.001	637 (5.4)	743 (18.3)	< 0.001
Solid tumor (*n*, %)	544 (3.4)	330 (2.4)	214 (10.3)	< 0.001	159 (1.3)	385 (9.5)	< 0.001
AIDS (*n*, %)	30 (0.2)	27 (0.2)	3 (0.1)	0.79	22 (0.2)	8 (0.2)	0.884
Treatment							
Aspirin (*n*, %)	8,105 (50.9)	7,339 (53)	766 (36.8)	< 0.001	6,585 (55.5)	1,520 (37.4)	< 0.001
Heparin (*n*, %)	6,729 (42.2)	5,372 (38.8)	1,357 (65.3)	< 0.001	4,286 (36.1)	2,443 (60.1)	< 0.001
Statin (*n*, %)	6,458 (40.5)	5,796 (41.8)	662 (31.8)	< 0.001	5,148 (43.4)	1,310 (32.2)	< 0.001
Vasoactive agent (*n*, %)	7,309 (45.9)	6,201 (44.8)	1,108 (53.3)	< 0.001	5,465 (46.1)	1,844 (45.4)	0.462
CRRT (*n*, %)	651 (4.1)	351 (2.5)	300 (14.4)	< 0.001	229 (1.9)	422 (10.4)	< 0.001
Mechanical ventilation (*n*, %)	1,333 (8.4)	1,149 (8.3)	184 (8.9)	0.394	910 (7.7)	423 (10.4)	< 0.001
Length of stay (LOS)							
LOS in hospital	7.4 (5.0, 11.9)	7.5 (5.1, 11.8)	7.0 (3.6, 12.6)	< 0.001	7.1 (5.0, 10.8)	8.7 (4.9, 15.6)	< 0.001
LOS in ICU	2.2 (1.4, 4.0)	2.1 (1.3, 3.7)	3.6 (1.9, 6.7)	< 0.001	2.1 (1.3, 3.5)	3.1 (1.9, 6.1)	< 0.001

SBP, systolic blood pressure; DBP, diastolic blood pressure; MAP, mean arterial pressure; HCT, hematocrit; Hb, hemoglobin; PLT, platelets; INR, international normalized ratio; PT, prothrombin time; PTT, partial thromboplastin time; CRRT, continuous renal replacement therapy.

### 3.2 Feature selection

#### 3.2.1 LASSO regression

**Figure 2:**
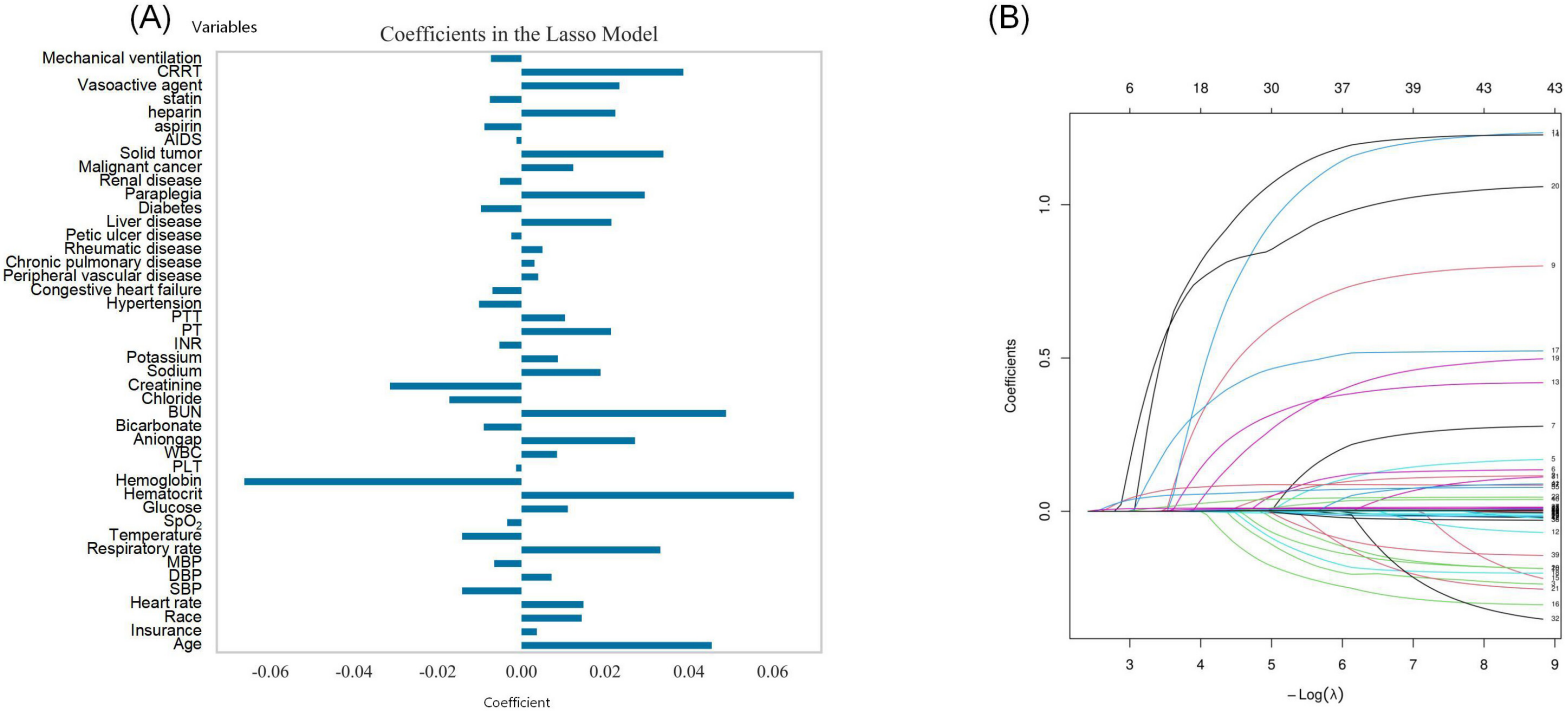
Preliminary screening features of 28-day mortality risk: **(A)** Feature selection using Lasso regression algorithm; **(B)** The variation characteristics of variable coefficients.

A total of 44 key features for predicting 28-day mortality risk were identified via LASSO regression analysis, as outlined in Figure 2A in descending order of feature importance: age, insurance type, race, heart rate, SBP, DBP, MAP, respiratory rate, temperature, SpO2, glucose, hematocrit, hemoglobin, platelet count, WBC, anion gap, bicarbonate, BUN, chloride, creatinine, sodium, potassium, INR, PT, PTT, hypertension, congestive heart failure, peripheral vascular disease, chronic pulmonary disease, rheumatic disease, peptic ulcer disease, liver disease, diabetes, paraplegia, renal disease, malignant cancer, solid tumors, AIDS, and exposure to aspirin, heparin, statins, vasoactive agents, CRRT, and mechanical ventilation. The distribution of regression coefficients for these variables is presented in Figure 2B.

**Figure 3:**
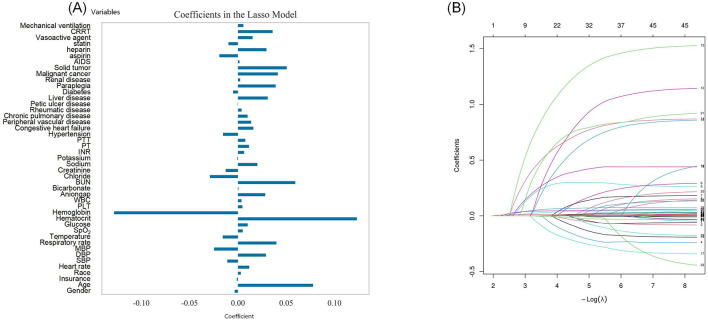
Preliminary screening features of 365-day mortality risk: **(A)** Feature selection using Lasso regression algorithm; **(B)** The variation characteristics of variable coefficients.

Using the identical LASSO regression approach, 45 important features were identified for the 365-day mortality risk prediction model—with the addition of the gender variable relative to the 28-day model (Figures 3A, B).

#### 3.2.2 Diagnosis of collinearity

To evaluate the presence of multicollinearity among the 44 features in the 28-day mortality risk prediction model, VIF was employed for collinearity diagnosis. As presented in Table 2, Hematocrit, Hemoglobin, MAP, DBP, PT, and INR had VIF values exceeding five—indicating strong multicollinearity. To address this, the following feature selection strategy was implemented: Hematocrit and Hemoglobin were highly correlated; given that Hemoglobin is more widely utilized in clinical risk stratification, Hemoglobin was retained while Hematocrit was excluded; MAP and DBP are both blood pressure-related metrics. Since SBP exhibits higher variable independence and was already included in the model, MAP and DBP were excluded; PT and INR both reflect coagulation function and are strongly associated with PTT. However, PTT showed no significant multicollinearity; thus, PTT was retained, and PT/INR were excluded.

**Table 2:** Collinearity diagnostic results of the 28-day mortality risk prediction model.

Variables	VIF	Variables	VIF
Hematocrit	19.008	Potassium	1.279
Hemoglobin	18.507	Solid tumor	1.272
MAP	9.281	SpO2	1.265
PT	8.323	CRRT	1.235
INR	8.219	Diabetes	1.217
DBP	6.561	Platelets	1.202
SBP	3.198	PTT	1.196
Chloride	2.738	Aspirin	1.154
BUN	2.445	Statin	1.143
Creatinine	2.365	Temperature	1.126
Sodium	2.118	WBC	1.114
Aniongap	1.861	Chronic pulmonary disease	1.093
Renal disease	1.8	Liver disease	1.082
Bicarbonate	1.754	Paraplegia	1.077
Age	1.751	Peripheral vascular disease	1.048
Glucose	1.619	Peptic ulcer disease	1.042
Hypertension	1.506	Race	1.039
Insurance	1.473	Mechanical ventilation	1.029
Heart rate	1.393	Rheumatic disease	1.013
Respiratory rate	1.366	AIDS	1.009
Heparin	1.359		
Congestive heart failure	1.332		
Vasoactive agent	1.312		
Malignant cancer	1.291		

For the 365-day mortality risk prediction model, collinearity analysis results were largely consistent with those of the 28-day model. Accordingly, features were optimized following the identical selection criteria, with detailed results provided in Table 3.

**Table 3:** Collinearity diagnostic results of the 365-day mortality risk prediction model.

Variables	VIF	Variables	VIF
Hematocrit	19.168	Potassium	1.29
Hemoglobin	18.866	Solid tumor	1.272
MAP	9.283	SpO2	1.265
PT	8.327	CRRT	1.235
INR	8.22	Diabetes	1.217
DBP	6.584	Platelets	1.216
SBP	3.206	PTT	1.196
Chloride	2.739	Gender	1.179
BUN	2.449	Aspirin	1.157
Creatinine	2.379	Statin	1.144
Sodium	2.119	Temperature	1.128
Aniongap	1.873	WBC	1.114
Renal disease	1.803	Chronic pulmonary disease	1.099
Bicarbonate	1.755	Liver disease	1.082
Age	1.754	Paraplegia	1.078
Glucose	1.619	Peripheral vascular disease	1.048
Hypertension	1.507	Peptic ulcer disease	1.043
Insurance	1.475	Race	1.039
Heart rate	1.4	Mechanical ventilation	1.03
Respiratory rate	1.366	Rheumatic disease	1.021
Heparin	1.364	AIDS	1.01
Congestive heart failure	1.334		
Vasoactive agent	1.314		
Malignant cancer	1.296		

### 3.3 Machine learning construction and comparison

**Figure 4:**
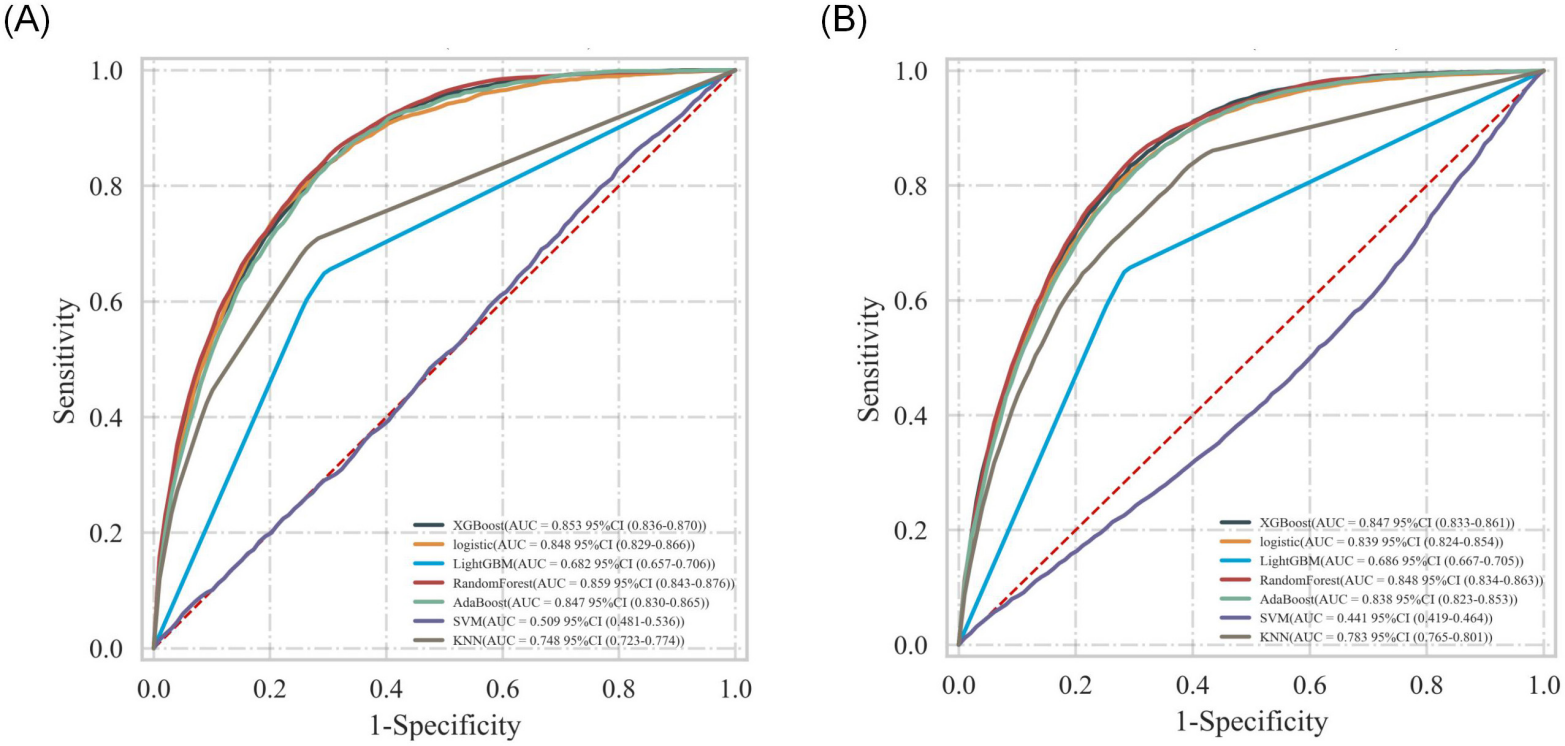
Comparison of ROC curves in training sets of multiple algorithms: **(A)** 28-day mortality risk; **(B)** 365-day mortality risk.

To evaluate and select the optimal algorithm among seven machine learning models, five-fold cross-validation was employed, with performance assessed using metrics including area under the receiver operating characteristic curve (AUC-ROC), accuracy, and F1-score. The discriminative performance of the seven models in terms of ROC curves is visualized in Figures 4A, B, while detailed performance metrics are presented in Tables 4 and 5. Results indicated that RandomForest achieved the optimal predictive performance for both outcomes: For 28-day mortality risk: AUC-ROC = 0.859 (95% CI: 0.843–0.876), accuracy = 0.88 (95% CI: 0.879–0.881); For 365-day mortality risk: AUC-ROC = 0.848 (95% CI: 0.834–0.863), accuracy = 0.798 (95% CI: 0.791–0.805).

**Table 4:** Comparison of 28-day mortality risk prediction performance of different algorithms on the validation set.

Algorithm	AUC (95% CI)	Accuracy (95% CI)	Sensitivity (95% CI)	Specificity (95% CI)	F1 score (95% CI)
XGBoost	0.853 (0.836–0.870)	0.774 (0.766–0.783)	0.747 (0.706–0.789)	0.778 (0.766–0.791)	0.464 (0.448–0.479)
logistic	0.848 (0.829–0.866)	0.762 (0.750–0.773)	0.776 (0.757–0.796)	0.76 (0.744–0.775)	0.46 (0.452–0.467)
LightGBM	0.682 (0.657–0.706)	0.71 (0.698–0.722)	0.644 (0.616–0.672)	0.72 (0.704–0.735)	0.367 (0.355–0.378)
RandomForest	0.859 (0.843–0.876)	0.88 (0.879–0.881)	0.136 (0.115–0.157)	0.992 (0.990–0.994)	0.228 (0.199–0.257)
AdaBoost	0.847 (0.830–0.865)	0.736 (0.729–0.743)	0.801 (0.755–0.848)	0.726 (0.712–0.741)	0.442 (0.432–0.451)
SVM	0.509 (0.481–0.536)	0.435 (0.207–0.663)	0.711 (0.376–1.047)	0.394 (0.083–0.705)	0.219 (0.140–0.298)
KNN	0.748 (0.723–0.774)	0.727 (0.721–0.734)	0.703 (0.676–0.731)	0.731 (0.721–0.741)	0.402 (0.395–0.409)

**Table 5:** Comparison of 365-day mortality risk prediction performance of different algorithms on the validation set.

Algorithm	AUC (95% CI)	Accuracy (95% CI)	Sensitivity (95% CI)	Specificity (95% CI)	F1 score (95% CI)
XGBoost	0.847 (0.833–0.861)	0.761 (0.751–0.771)	0.781 (0.770–0.792)	0.755 (0.740–0.770)	0.625 (0.616–0.635)
logistic	0.839 (0.824–0.854)	0.736 (0.725–0.747)	0.823 (0.800–0.847)	0.706 (0.688–0.724)	0.614 (0.603–0.625)
LightGBM	0.686 (0.667–0.705)	0.704 (0.697–0.711)	0.649 (0.617–0.681)	0.723 (0.709–0.736)	0.528 (0.514–0.542)
RandomForest	0.848 (0.834–0.863)	0.798 (0.791–0.805)	0.464 (0.386–0.543)	0.912 (0.882–0.943)	0.536 (0.493–0.579)
AdaBoost	0.838 (0.823–0.853)	0.733 (0.712–0.753)	0.815 (0.792–0.839)	0.704 (0.669–0.740)	0.609 (0.597–0.621)
SVM	0.441 (0.419–0.464)	0.477 (0.273–0.681)	0.584 (0.139–1.030)	0.441 (0.014–0.867)	0.268 (0.094–0.442)
KNN	0.783 (0.765–0.801)	0.751 (0.749–0.752)	0.032 (0.027–0.037)	0.997 (0.996–0.998)	0.061 (0.052–0.071)

**Figure 5:**
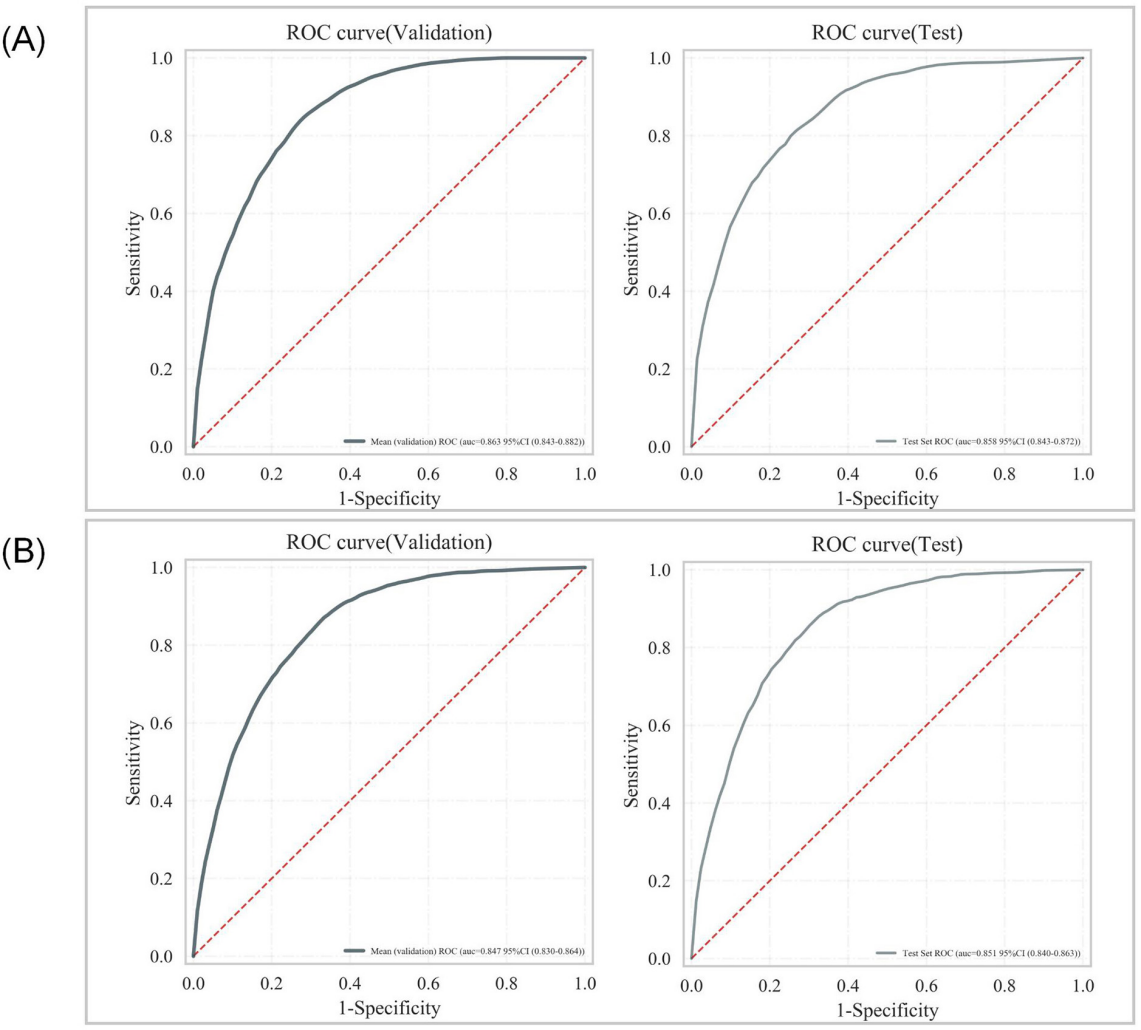
Internal validation ROC curve for the random forest model: **(A)** 28-day mortality risk; **(B)** 365-day mortality risk.

For internal validation, the dataset was partitioned into a training set and an independent test set at a 7:3 ratio. Five-fold cross-validation was performed on the training set, and grid search was used for hyperparameter tuning to identify the optimal hyperparameter configuration. Results demonstrated that RandomForest retained excellent mortality risk prediction performance on the test set (Figures 5A, B).

**Figure 6:**
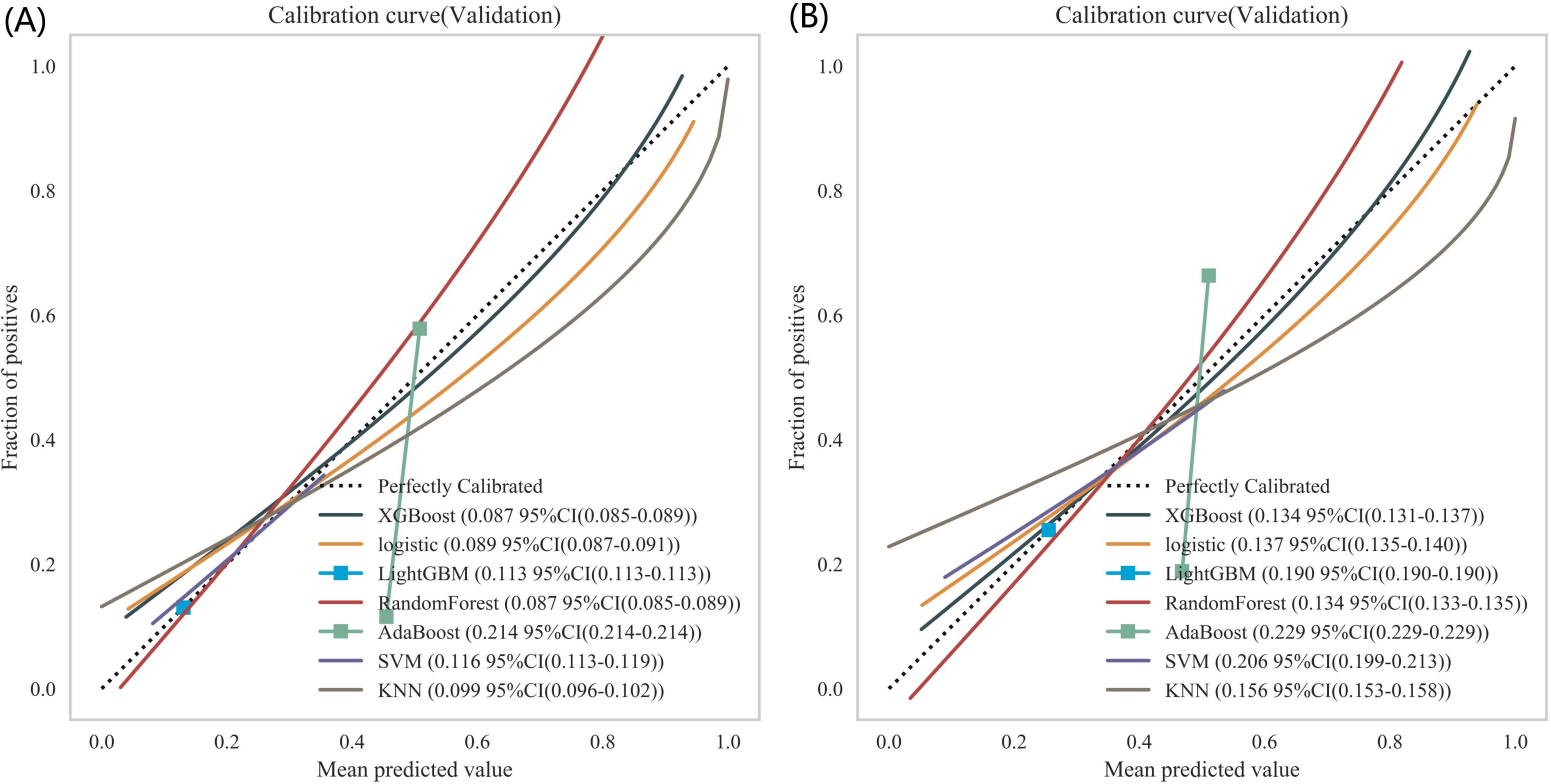
The calibration curves of the respective models: **(A)** 28-day mortality risk; **(B)** 365-day mortality risk.

The calibration curves of the respective models are presented in Figures 6A, B. For short-term prognosis prediction, the XGBoost, logistic regression, and random forest models exhibited the optimal and comparable calibration accuracy, with a uniform CE of 0.087. In long-term prognosis prediction, the CE of all models increased as expected: XGBoost and random forest remained the top-performing models (both with a CE of 0.134), while the AdaBoost model yielded the largest CE (0.229). Given that a CE < 0.2 is generally recognized as a favorable metric for clinical prediction models, these results indicate that the accuracy of the models' probability predictions falls within an acceptable range. The calibration plots of the standalone random forest model (Supplementary Figure 1) revealed the presence of systematic bias, particularly overconfidence in the high-risk region. To enhance the interpretability of predicted probabilities, we implemented two *post-hoc* calibration methods (Supplementary Table 2): Platt scaling and isotonic regression. Following calibration, the Brier score for 28-day mortality prediction decreased to 0.210 (Platt scaling) and 0.199 (isotonic regression), respectively; for 365-day mortality prediction, the Brier score reduced to approximately 0.210. Calibration curves showed a marked improvement in the agreement between predicted probabilities and actual event rates after calibration. Integrating the findings from both time points, the random forest model demonstrated the most superior and stable calibration performance while maintaining high discriminative ability, with the strongest agreement between predicted probabilities and observed risks.

Given that the 28-day mortality rate is only approximately 13%, the use of the default probability threshold of 0.5 may underestimate the actual discriminative capability of the model. Therefore, we performed a threshold sensitivity analysis focusing on the F1 score (Supplementary Table 3). For the 28-day mortality prediction, the optimal threshold derived via F1-score optimization was 0.04. At this threshold, the sensitivity increased to 0.636, the specificity was 0.527, and the F1-score improved to 0.464. For the 365-day mortality prediction, the F1-optimal threshold was 0.01, at which the sensitivity reached 0.750, suggesting that the model also exhibits robust high-risk capture ability in long-term risk identification. The results indicate that the random forest model can achieve a more rational balance between sensitivity and specificity following appropriate threshold adjustment. Due to class imbalance may lead to overestimation of model performance by the ROC curve, we generated the precision-recall (PR) curve (Supplementary Figure 2). For the 28-day mortality prediction, the area under the PR curve (AUPRC) was 0.355; for the 365-day mortality prediction, the AUPRC was 0.300, both of which were significantly higher than the random baseline. This further demonstrates that the model retains robust risk identification capability in the context of imbalanced data.

**Figure 7:**
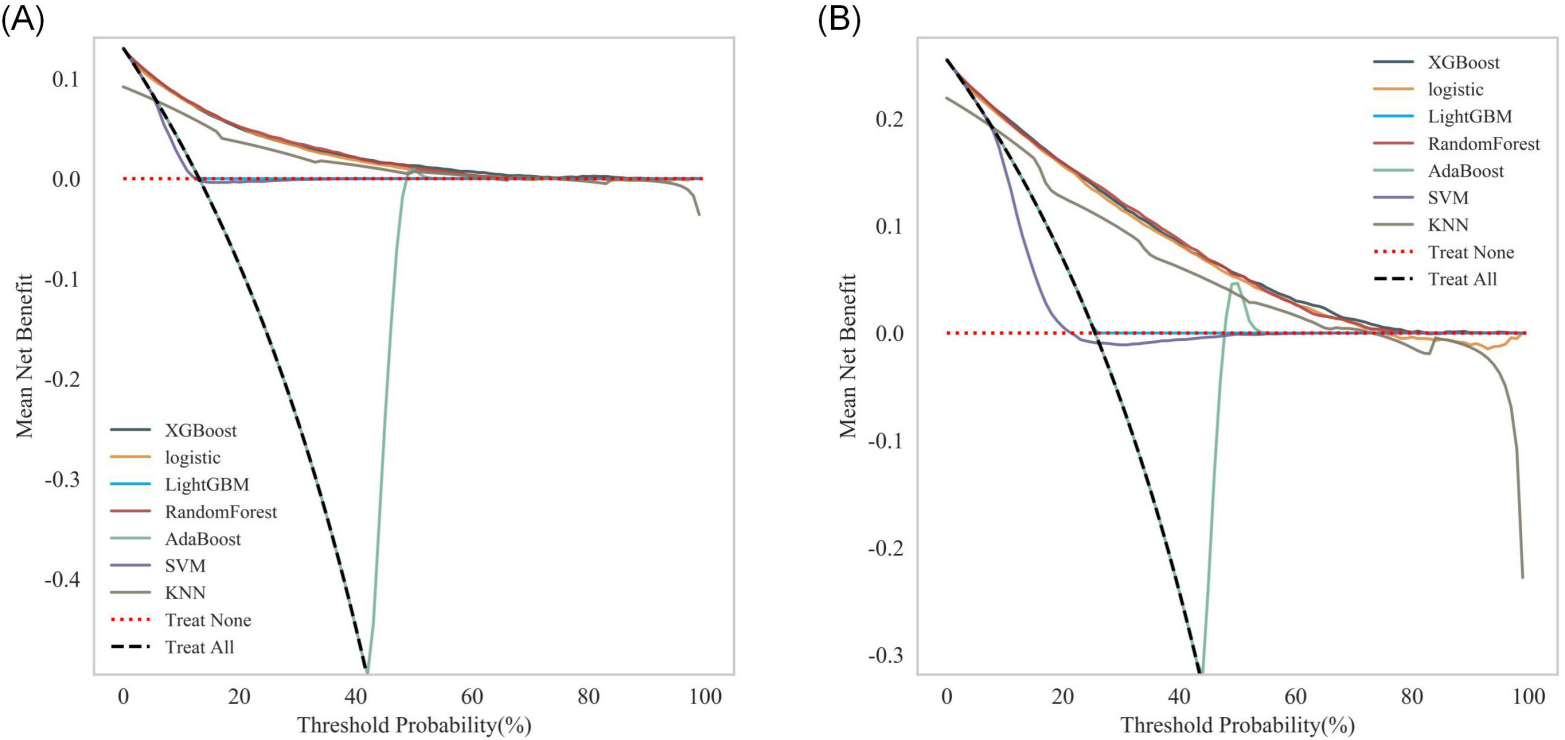
Decision analysis of the respective models: **(A)** 28-day mortality risk; **(B)** 365-day mortality risk.

In addition, within the DCA, the decision curve of the Random Forest model was overall smooth, exhibiting robust adaptability to threshold variations (Figures 7A, B). This characteristic allowed the model to sustain a high net benefit across clinical scenarios with diverse risk preferences. Combined with the model's favorable discriminative ability, acceptable calibration, and robust net benefit performance, it demonstrates potential for translation into a clinical decision-support tool—though further validation in prospective studies remains warranted.

### 3.4 Interpretable analysis

#### 3.4.1 Prediction model for 28-day mortality risk

**Figure 8:**
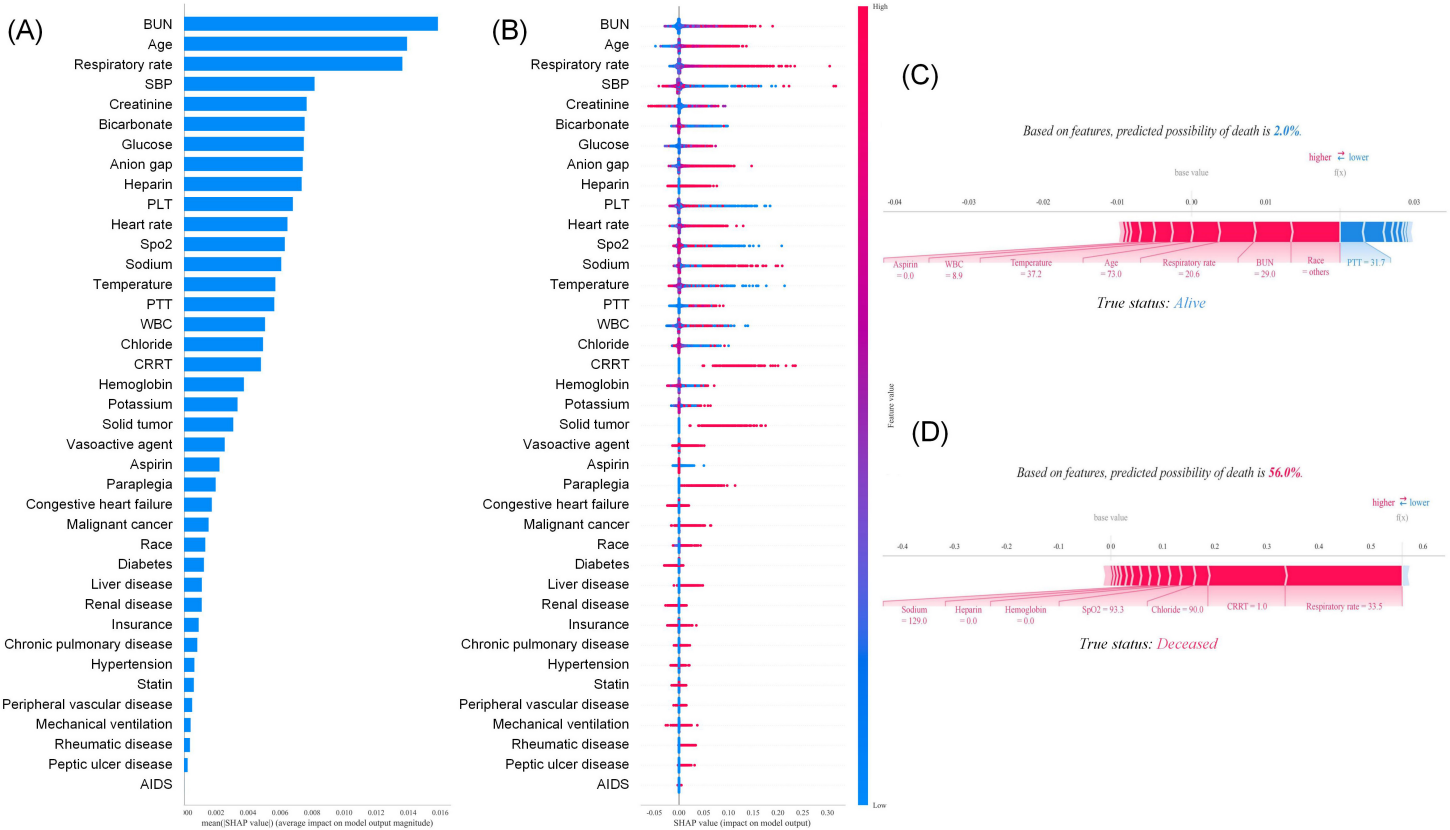
Interpretation of 28-day mortality risk prediction model based on SHAP method: **(A)** Summary diagram; **(B)** feature importance map; **(C)** The individual force plot of an survivor; **(D)** The individual force plot of an non-survivor.

The SHAP feature importance plot (Figure 8A) illustrates the magnitude of feature importance contributions; Figure 8B displays the distribution of SHAP values for each feature, where the influence of SHAP values decreases sequentially from top to bottom. The ranking of features is consistent between the two plots, with key features including BUN, age, SBP, respiratory rate, creatinine, bicarbonate, glucose, anion gap, warfarin exposure, and platelet count. For the 28-day mortality risk prediction model, SHAP explanation plots revealed that a surviving patient had a predicted mortality probability of 2% (Figure 8C), which aligned with their true label (survival), confirming the accuracy of the prediction. Key features contributing to an elevated 28-day mortality risk in this case included higher blood urea nitrogen levels, an increased respiratory rate, and advanced age. In contrast, a deceased patient exhibited a predicted mortality probability of 56.0% (Figure 8D), which was consistent with their true label (death). This further enhances the model's interpretability and transparency. For this patient, the critical driving factors behind the elevated 28-day mortality risk were an increased respiratory rate and the severe clinical status indicated by the implementation of CRRT.

#### 3.4.2 Prediction model for 365-day mortality risk

**Figure 9:**
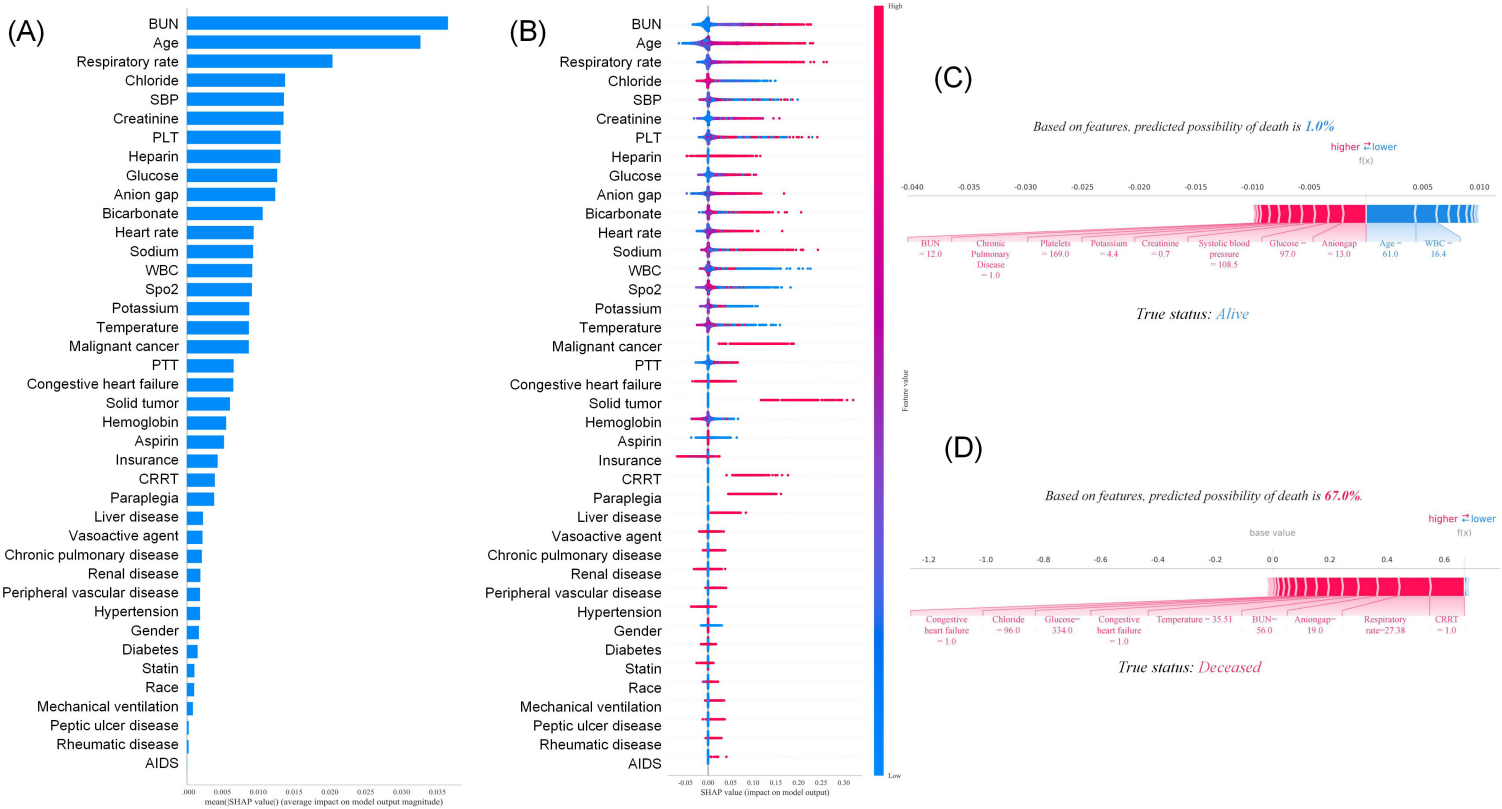
Interpretation of 365-day mortality risk prediction model based on SHAP method: **(A)** Summary diagram; **(B)** feature importance map; **(C)** The individual force plot of an survivor; **(D)** The individual force plot of an non-survivor.

The SHAP feature importance plot (Figure 9A) depicts the contribution magnitude of feature importance; Figure 9B presents the distribution of SHAP values for each feature, with SHAP influence declining in descending order from top to bottom. The feature ranking is consistent across both plots, encompassing BUN, age, respiratory rate, chloride, SBP, creatinine, platelet count, warfarin exposure, glucose, anion gap, bicarbonate, and heart rate. For the 365-day mortality risk prediction model, SHAP explanation plots demonstrated that a surviving patient had a predicted mortality probability of 1.0% (Figure 9C), which was consistent with their true label (survival), thus confirming the model's predictive accuracy. Conversely, a deceased patient exhibited a predicted mortality probability of 67.0% (Figure 9D), which aligned with their true label (death). This further enhances the model's interpretability and transparency. For this deceased patient, the primary drivers of the elevated 365-day mortality risk included severe clinical status indicated by the implementation of continuous renal replacement therapy (CRRT), an increased respiratory rate, and a higher anion gap level.

To enhance the clinical interpretability of the model's explanatory results, we summarized the top 10 key predictive variables identified by SHAP analysis for each of the two outcomes, and presented their risk directions along with corresponding clinical interpretations (Supplementary Tables 4 and 5).

### 3.5 Sensitivity analysis

To evaluate the stability of the model in a more homogeneous clinical population, we restricted the study cohort to patients with acute myocardial infarction (ICD-10: I21, I22) and performed a sensitivity analysis (Supplementary Figure 3). For 28-day mortality prediction: the AUCs of the models ranged from 0.809 to 0.829. The random forest model achieved an AUC of 0.828 (95% CI: 0.791–0.866), which was comparable to the results observed in the overall CAD population. For 365-day mortality prediction: the AUCs of the models spanned 0.776 to 0.839. The random forest model demonstrated an AUC of 0.839 (95% CI: 0.808–0.870), retaining robust discriminatory performance. These findings indicate that the random forest model maintains stable performance in the more homogeneous AMI subgroup.

### 3.6 External validation

**Figure 10:**
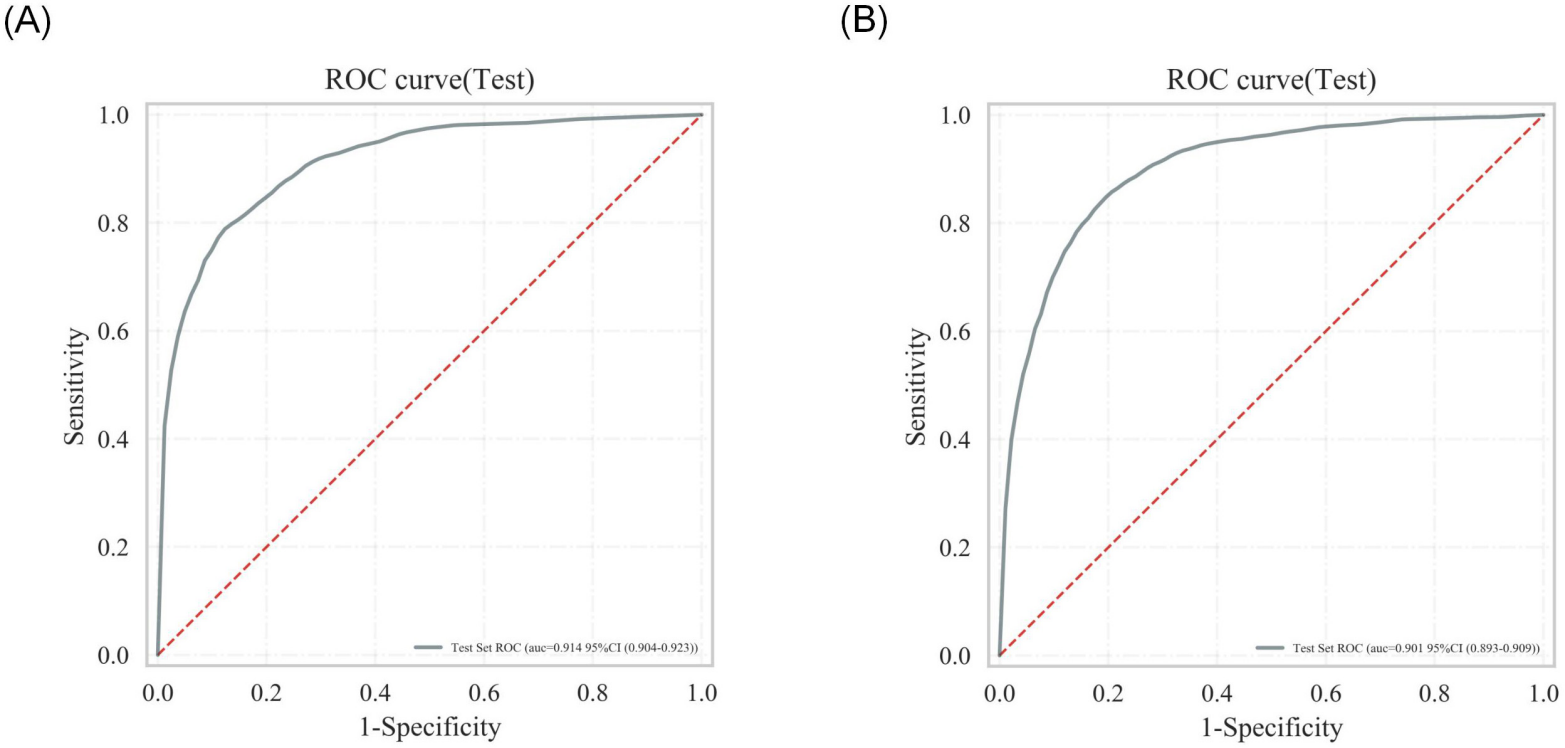
External validation ROC curve for the random forest model: **(A)** 28-day mortality risk; **(B)** 365-day mortality risk.

In line with the consistent inclusion and exclusion criteria specified earlier (Figure 1), data of patients with CAD were extracted from the MIMIC-III database to establish an external validation cohort. Results indicated that the optimal model—the Random Forest algorithm—retained excellent discriminative performance in predicting 28- and 365-day mortality risks, while demonstrating robust generalization capability to independent external datasets. As visualized in Figures 10A, B, the AUC-ROC exceeded 0.9 for both outcomes, further validating the model's stability and reliability [28-day mortality risk: AUC = 0.914, 95% CI: 0.904–0.923; 365-day mortality risk: AUC = 0.901, 95% CI: 0.893–0.909].

## 4 Discussion

In patients with coronary artery disease admitted to the ICU, clinical manifestations are often characterized by complexity and rapid progression. The early and accurate identification of high-risk individuals is of pivotal significance for improving prognosis; particularly in critical scenarios such as acute myocardial infarction, timely implementation of interventional measures can markedly mitigate mortality risk (14). Traditional risk scoring systems or single-variable-based prediction methods typically exhibit suboptimal predictive performance and inadequate interpretability, which constrains their widespread utility in clinical practice. Fully black-box models struggle to gain clinical endorsement, whereas models with artificially enhanced interpretability may compromise predictive accuracy (15). In recent years, advanced machine learning methodologies have demonstrated robust capabilities in processing high-dimensional and nonlinear data, offering novel technical avenues for clinical risk assessment—for instance, enhancing predictive precision by mining complex association patterns within multi-center electronic health records (16). Nevertheless, there remains a paucity of practical prediction tools that integrate routine clinical indicators, have undergone independent external validation, and possess favorable interpretability (9). The present study employs a variety of machine learning algorithms to construct risk prediction models, screens for core features, and incorporates interpretability analysis approaches to enhance the model's transparency and clinical credibility.

Given the significant multicollinearity between MAP and SBP, SBP—with a more stable predictive contribution—was retained following LASSO regularization and multicollinearity diagnostics. While MAP holds critical physiological relevance in the ICU, SBP is more ubiquitously documented as a discrete variable in electronic health records (EHRs) and exhibits a more well-established epidemiological association with cardiovascular outcomes. Furthermore, re-modeling with MAP substituted for SBP yielded no significant improvement in the model's discriminative performance (AUC; Supplementary Figure 4). The retention of PTT likely reflects the prevalence of heparin anticoagulation within the ICU cohort of this study. These variable selection choices may compromise the model's external generalizability, particularly in clinical settings where MAP and INR are preferred metrics.

When predicting 28- and 365-day mortality in critically ill patients with CAD, the Random Forest model exhibited the optimal performance among seven machine learning algorithms. Not only did this model yield the highest predictive accuracy, but it also demonstrated superior clinical net benefit in DCA compared to other algorithms, underscoring its value for clinical decision-making (17, 18). Its relatively high AUC value ensures robust risk stratification capability; the acceptable calibration (CE < 0.2) indicates that the output risk probabilities hold clinical reference value; and the subsequent DCA analysis further demonstrates that the model can yield meaningful clinical net benefits across a broad range of decision thresholds. While calibration analysis revealed a degree of bias in the probability outputs of the uncalibrated random forest model. Following Platt and Isotonic calibration, the Brier score decreased markedly and the reliability of the predicted probabilities improved significantly. This finding suggests that the raw probability outputs of the random forest model require calibration prior to clinical implementation. At the default threshold of 0.5, the model exhibited relatively low sensitivity in predicting 28-day mortality. Nevertheless, threshold optimization analysis based on the F1-score demonstrated a significant enhancement in sensitivity, indicating that for imbalanced datasets, adjusting the classification threshold enables flexible adaptation of the model to diverse clinical scenarios. Concurrently, in the more clinically homogeneous population with AMI, the model retained discriminatory performance comparable to that observed in the overall CAD cohort. External validation using the MIMIC-III database further confirmed the model's robustness and generalizability—a finding consistent with the results of Guan, Silvey, et al., (19, 20) whose Random Forest models maintained high discriminative performance across multi-center datasets. Considering the above aspects comprehensively, the random forest was selected as the final model on account of its superior overall performance. Through interpretability analysis via SHAP, this study identified key predictive features: core variables for short-term prognosis (28 days) included blood urea nitrogen, age, and systolic blood pressure, while blood urea nitrogen, age, and respiratory rate dominated long-term prognosis (365 days). These findings are highly congruent with the critical illness predictors identified using the SHAP method in studies by Yang, Bai, et al. (21, 22). The individualized interpretation function of SHAP force plots effectively enhanced model transparency, enabling clinicians to comprehend the prediction logic for specific patients. This study developed an early and late prognosis prediction system for critically ill patients with CAD based on the Random Forest model and SHAP methodology. The Random Forest model exhibited exceptional performance in prognostic prediction, leveraging its ensemble learning properties to effectively handle high-dimensional and nonlinear medical data while mitigating the risk of overfitting (23). SHAP enhances model interpretability by quantifying the contribution of each feature to the predictive outcome (24).

SHAP analysis revealed that 28-day mortality is primarily driven by factors such as acute physiological disturbance and insufficient organ perfusion, with BUN ranking first in contribution. Elevated BUN typically indicates reduced renal perfusion—e.g., decreased cardiac output or overactivation of the vasopressin system—leading to the accumulation of nitrogenous metabolites, exacerbation of internal environment imbalance, and reflection of early prerenal hypoperfusion (25). Second, age is a critical variable influencing short-term mortality risk. Aging is associated with vascular sclerosis, diminished compensatory function, and reduced multi-organ reserve capacity, rendering the body less tolerant to acute stress and thus contributing to elevated short-term mortality risk in critically ill patients (26). Respiratory rate, as a highly sensitive physiological parameter, also plays a significant role in short-term mortality prediction. Tachypnea often indicates a state of hypoxic compensation (e.g., cardiogenic pulmonary edema or metabolic acidosis), which reflects oxygen supply-demand imbalance and lactic acid accumulation—direct manifestations of disease severity (27). Furthermore, systolic blood pressure and creatinine collectively underscore the key role of cardiorenal interaction in critical illness. Low systolic blood pressure suggests cardiogenic shock or insufficient global effective circulating volume, leading to reduced glomerular filtration rate and elevated serum creatinine levels, forming a vicious cycle of “cardiorenal syndrome” (25, 28). Metabolically, alterations in bicarbonate and blood glucose further amplify short-term mortality risk. Low bicarbonate levels mark metabolic acidosis (e.g., lactic acidosis caused by insufficient tissue perfusion), which can rapidly inhibit myocardial contractility and worsen circulatory status (29). Hyperglycemia reflects enhanced stress response, promoting the development of multiple organ dysfunction by exacerbating endothelial injury, inducing immunosuppression, and driving inflammatory responses (28). These variables collectively constitute the core drivers of 28-day mortality risk in critically ill patients.

Compared with short-term mortality, 365-day mortality places greater emphasis on the cumulative effects of chronic organ damage and the persistent impacts of long-term metabolic and coagulation dysfunction. Beyond BUN, age, and respiratory rate—variables that retain extremely high contribution—internal environment-related indicators such as chloride ions and anion gap exert significant influence. Hypochloremia, commonly observed in states of excessive diuretic use or hypovolemia, can activate the renin-angiotensin system, further exacerbating electrolyte disturbances (30); an elevated anion gap indicates the accumulation of unmeasured acidic substances (e.g., increased uremic toxins), serving as a critical marker of long-term metabolic derangement and progressive renal dysfunction (24). Platelet count and warfarin use constitute another key component of long-term mortality risk. Thrombocytopenia may arise from consumptive processes in sepsis or drug-induced suppression, elevating bleeding risk (31); as an anticoagulant, warfarin is associated with an increased risk of severe bleeding or thrombotic events when dosage is poorly controlled, thereby compromising long-term survival (31). Additionally, persistent elevation of BUN and creatinine signifies the progression of chronic kidney disease (CKD), which significantly increases long-term mortality risk by promoting vascular calcification and accelerating atherosclerosis (25). Age and respiratory rate remain pivotal in long-term mortality. Elderly patients with reduced respiratory reserve are more susceptible to chronic hypoxemia, which induces myocardial ischemia, tachycardia, and disturbances in oxygen metabolism (32). Blood glucose and bicarbonate collectively reflect the impact of sustained metabolic burden on the body: hyperglycemia, indicative of insulin resistance and chronic inflammation, exacerbates vascular injury; acidosis, meanwhile, inhibits myocardial contractility and impairs neurohumoral regulatory capacity—both factors synergistically augment long-term cardiovascular mortality risk (28, 29). These variables collectively demonstrate that 365-day mortality is shaped by persistent organ dysfunction following acute events, long-term metabolic imbalance, and challenges in coagulation system regulation, mirroring the physiological vulnerability and rehabilitation hurdles faced by critically ill patients during the long-term recovery phase.

Consistent with multiple prior studies, the present research confirms the superior performance of the Random Forest model in analyzing complex clinical data, supporting its status as the optimal choice in multi-model comparisons and validating the significance of multi-algorithm evaluation for optimizing predictive performance. Additionally, this study not only continues the practice of external model validation established in previous research—demonstrating the model's robustness in independent populations—but also addresses the limitation of traditional studies that predominantly focus on short-term prognosis (e.g., predicting only 3- or 30-day mortality) by developing a dual-time-scale prognostic model covering both 28- and 365-day outcomes. Through the integration of the SHAP method, key predictors consistent with prior findings (e.g., age, renal function indices) were further identified, significantly enhancing the model's transparency and risk stratification capability. Notably, all these key variables are routine clinical indicators that are readily accessible in clinical settings.

## 5 Limitations and future prospects

Despite the favorable performance of this study in prediction accuracy and model interpretability, several limitations should be acknowledged: First, the data were derived from a retrospective design, which may introduce selection bias. Second, In contrast to time-to-event models, classification models fail to account for time-dependent variations in risk. Third, missing values for some key variables in the database could compromise the completeness and representativeness of the features. Concurrently, missing-data strategy with arbitrary thresholds for exclusion and KNN imputation may introduce bias. Fourth, the random forest algorithm employed in this study generates predictive probabilities based on the class proportion of training samples within leaf nodes, which may yield probabilities approaching extreme values (e.g., near 0 or 100%). While the overall calibration assessment (Brier Score) is favorable, this finding suggests that in clinical practice, post-processing calibration of output probabilities or the establishment of a lower threshold for risk reporting (e.g., classifying risks < 2% as “extremely low risk”) could be implemented to better align with the principle of clinical uncertainty and prevent potential misinterpretation. Fifth, Single-country, single health system dataset (US academic center) limits international generalizability. At the same time, possible misclassification of CAD from codes alone and the lack of ECG/imaging data. Lastly, although the model demonstrated good discrimination, calibration, and potential decision curve benefits, its ultimate clinical credibility and practical value must be established by validation in future independent, prospective clinical cohorts and by assessing its real impact on physician decision-making and patient outcomes when integrated into actual workflows.

Future research may encompass the following dimensions: (i) a prospective implementation study incorporating clinician feedback on SHAP-based explanations; (ii) comparison with or integration into established scoring systems (e.g., APACHE, SOFA) to demonstrate incremental clinical value; (iii) evaluation of model fairness and performance heterogeneity across clinically relevant subgroups (e.g., age, sex, race), given documented disparities in cardiovascular care; (iv) adoption of multi-center, prospective data collection strategies—coupled with dynamic time-series analysis methods (e.g., continuous physiological monitoring or longitudinal laboratory trajectory modeling)—to more authentically capture disease progression. Such efforts will enhance the model's robustness and clinical utility.

## 6 Conclusion

We developed a random forest prediction model with favorable performance and interpretability, based on the 28- and 365-day mortality risks of critically ill patients with CAD. This model exhibited relatively robust performance in external validation, offering a promising tool for the precise risk stratification of critically ill CAD patients. Furthermore, it lays a foundation for subsequent prospective clinical studies aimed at evaluating its real-world decision-making impact and effectiveness in improving patient outcomes.

## Data Availability

Publicly available datasets were analyzed in this study. This data can be found here: the clinical dataset used in this study is the Medical Information Mart for Intensive Care (MIMIC) database, available from PhysioNet, a research data repository hosted by the MIT Laboratory for Computational Physiology. Physiological and clinical data from intensive care unit stays were obtained under a data use agreement with PhysioNet (https://physionet.org/content/mimiciv/3.1/).
