# Supplementary material for: Interpretable machine learning for prognostic prediction in critically ill patients with coronary artery disease: a multicenter study
Source: Front Med (Lausanne). 2026 Mar 30;13:1794827. doi: 10.3389/fmed.2026.1794827 (PMC13070936; doi:10.3389/fmed.2026.1794827)
Supplement: Supplementary file 1 [file Data_Sheet_1.docx]

Supplementary Material

# Supplementary Figures and Tables

## Supplementary Figures

**
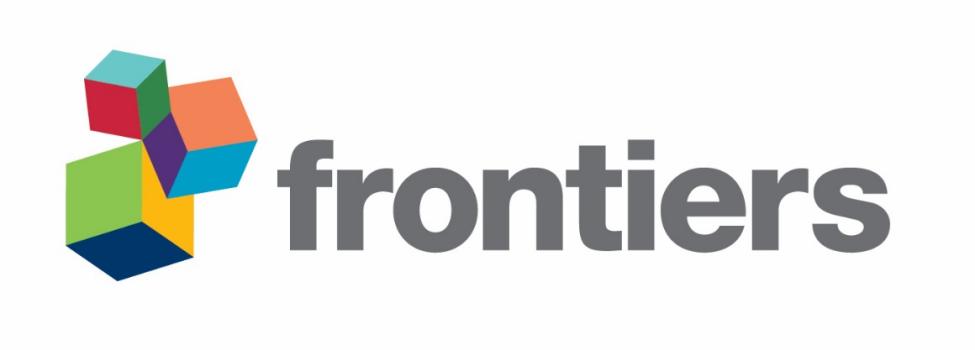
**

**Supplementary Figure 1.** Calibration plots of the Random Forest model for predicting 28-day and 365-day mortality. (A) Calibration plot for 28-day mortality; (B) Calibration plot for 365-day mortality

**Supplementary Figure 2.** Precision–recall (PR) curves of the Random Forest model for predicting 28-day and 365-day mortality. (A) PR curve for 28-day mortality; (B) PR curve for 365-day mortality

**Supplementary Figure 3.** Sensitivity analysis restricted to acute myocardial infarction (AMI) patients. (A) Model comparison for predicting 28-day mortality in AMI patients; (B) Model comparison for predicting 365-day mortality in AMI patients

**Supplementary Figure 4.** Sensitivity analysis replacing systolic blood pressure with mean arterial pressure. (A) 28-day mortality; (B) 365-day mortality

## Supplementary Tables

**Supplementary Table 1.** ICD Codes for Coronary Artery Disease

| **ICD Version** | **Code** |
| --- | --- |
| ICD-9 | 410.x |
| ICD-9 | 411.x |
| ICD-9 | 412 |
| ICD-9 | 413 |
| ICD-9 | 414 |
| ICD-10 | I20 |
| ICD-10 | I21 |
| ICD-10 | I22 |
| ICD-10 | I25 |

**Supplementary Table 2.** Calibration performance of the model before and after probability calibration

| **Model** | **Brier score** | **Model** | **Brier score** |
| --- | --- | --- | --- |
| **28-day mortality** |  | **365-day mortality** |  |
| Uncalibrated | 0.286 | Uncalibrated | 0.286 |
| Platt | 0.21 | Platt | 0.21 |
| Isotonic | 0.199 | Isotonic | 0.21 |

**Supplementary Table 3.** Threshold-based performance metrics for predicting 28-day and 365-day mortality

|  | **Threshold** | **Sensitivity** | **Specificity** | **Precision** | **F1** | **Balanced Accuracy** |
| --- | --- | --- | --- | --- | --- | --- |
| **28-day mortality** |  |  |  |  |  |  |
|  | 0.01 | 0.813 | 0.274 | 0.324 | 0.464 | 0.544 |
|  | 0.02 | 0.731 | 0.385 | 0.337 | 0.462 | 0.558 |
|  | 0.03 | 0.680 | 0.462 | 0.351 | 0.463 | 0.571 |
|  | 0.04 | 0.636 | 0.527 | 0.365 | 0.464 | 0.581 |
|  | 0.05 | 0.598 | 0.577 | 0.378 | 0.463 | 0.588 |
|  | 0.1 | 0.463 | 0.755 | 0.448 | 0.455 | 0.600 |
| **365-day mortality** |  |  |  |  |  |  |
|  | 0.01 | 0.750 | 0.247 | 0.300 | 0.428 | 0.498 |
|  | 0.02 | 0.649 | 0.350 | 0.300 | 0.410 | 0.500 |
|  | 0.03 | 0.578 | 0.419 | 0.299 | 0.394 | 0.498 |
|  | 0.04 | 0.522 | 0.478 | 0.3009 | 0.381 | 0.500 |
|  | 0.05 | 0.478 | 0.526 | 0.302 | 0.370 | 0.502 |
|  | 0.1 | 0.307 | 0.689 | 0.297 | 0.302 | 0.498 |

**Supplementary Table 4.** Top 10 SHAP predictors for 28-day mortality

| **Rank** | **Feature** | **Direction of Effect** | **Clinical Interpretation** |
| --- | --- | --- | --- |
| 1 | BUN | Higher values associated with increased risk | Elevated BUN reflects renal dysfunction and impaired tissue perfusion, which are strongly associated with poor outcomes in critically ill patients. |
| 2 | Age | Higher values associated with increased risk | Advanced age indicates reduced physiological reserve and increased burden of comorbidities. |
| 3 | Respiratory rate | Higher values associated with increased risk | Tachypnea may reflect respiratory distress, metabolic acidosis, or systemic deterioration. |
| 4 | Systolic blood pressure | Lower values associated with increased risk | Hypotension suggests hemodynamic instability and possible cardiogenic shock. |
| 5 | Creatinine | Higher values associated with increased risk | Elevated creatinine indicates kidney injury and multi-organ dysfunction. |
| 6 | Bicarbonate | Lower values associated with increased risk | Reduced bicarbonate indicates metabolic acidosis and severe physiological stress. |
| 7 | Glucose | Higher values associated with increased risk | Stress hyperglycemia reflects metabolic dysregulation and inflammatory response. |
| 8 | Anion gap | Higher values associated with increased risk | Elevated anion gap suggests metabolic acidosis and tissue hypoperfusion. |
| 9 | Heparin exposure | Presence associated with increased risk | Anticoagulation use may reflect greater illness severity or thrombotic risk requiring intensive treatment. |
| 10 | Platelet count | Lower values associated with increased risk | Thrombocytopenia may indicate systemic inflammation, coagulopathy, or severe illness. |

**Supplementary Table 5.** Top 10 SHAP predictors for 365-day mortality

| **Rank** | **Feature** | **Direction of Effect** | **Clinical Interpretation** |
| --- | --- | --- | --- |
| 1 | BUN | Higher values associated with increased risk | Chronic renal dysfunction and impaired perfusion are associated with long-term mortality. |
| 2 | Age | Higher values associated with increased risk | Older age reflects accumulated comorbidity burden and reduced long-term physiological resilience. |
| 3 | Respiratory rate | Higher values associated with increased risk | Persistent tachypnea may indicate cardiopulmonary dysfunction and systemic disease severity. |
| 4 | Chloride | Abnormal values associated with increased risk | Chloride imbalance may reflect metabolic disturbances and acid-base dysregulation. |
| 5 | Systolic blood pressure | Lower values associated with increased risk | Lower SBP indicates hemodynamic compromise and cardiovascular instability. |
| 6 | Creatinine | Higher values associated with increased risk | Chronic kidney dysfunction is a strong predictor of long-term mortality. |
| 7 | Platelet count | Lower values associated with increased risk | Thrombocytopenia may reflect chronic inflammation or systemic illness. |
| 8 | Heparin exposure | Presence associated with increased risk | Anticoagulation therapy may indicate higher cardiovascular or thrombotic risk. |
| 9 | Glucose | Higher values associated with increased risk | Hyperglycemia is associated with metabolic dysfunction and worse long-term outcomes. |
| 10 | Anion gap | Higher values associated with increased risk | Elevated anion gap reflects metabolic acidosis and systemic metabolic derangement. |
