# Supplementary figures and images for: Interpretable machine learning for prognostic prediction in critically ill patients with coronary artery disease: a multicenter study
Source: Front Med (Lausanne). 2026 Mar 30;13:1794827. doi: 10.3389/fmed.2026.1794827 (PMC13070936; doi:10.3389/fmed.2026.1794827)

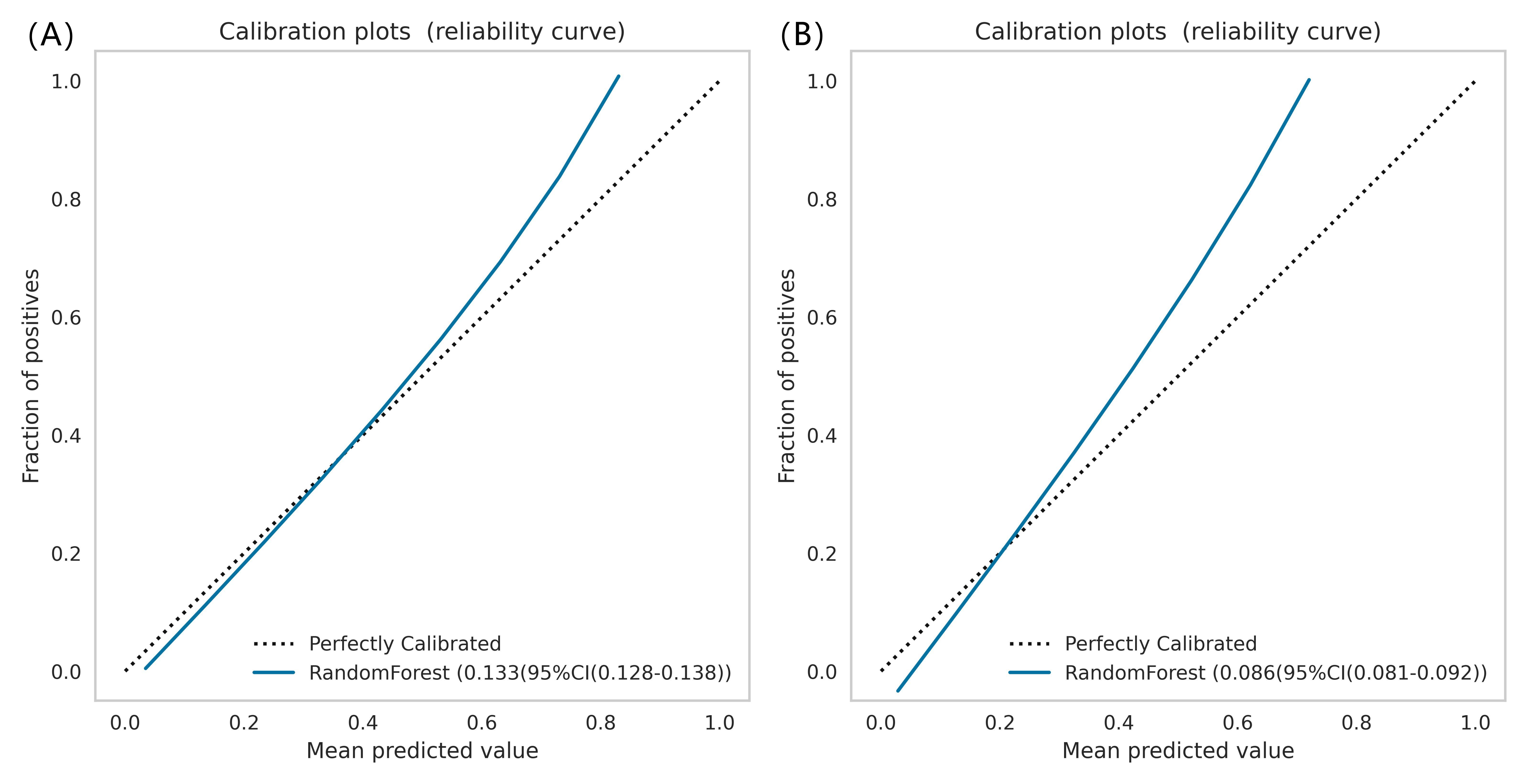

Supplement: Supplementary file 2 [file Image_1.jpeg]

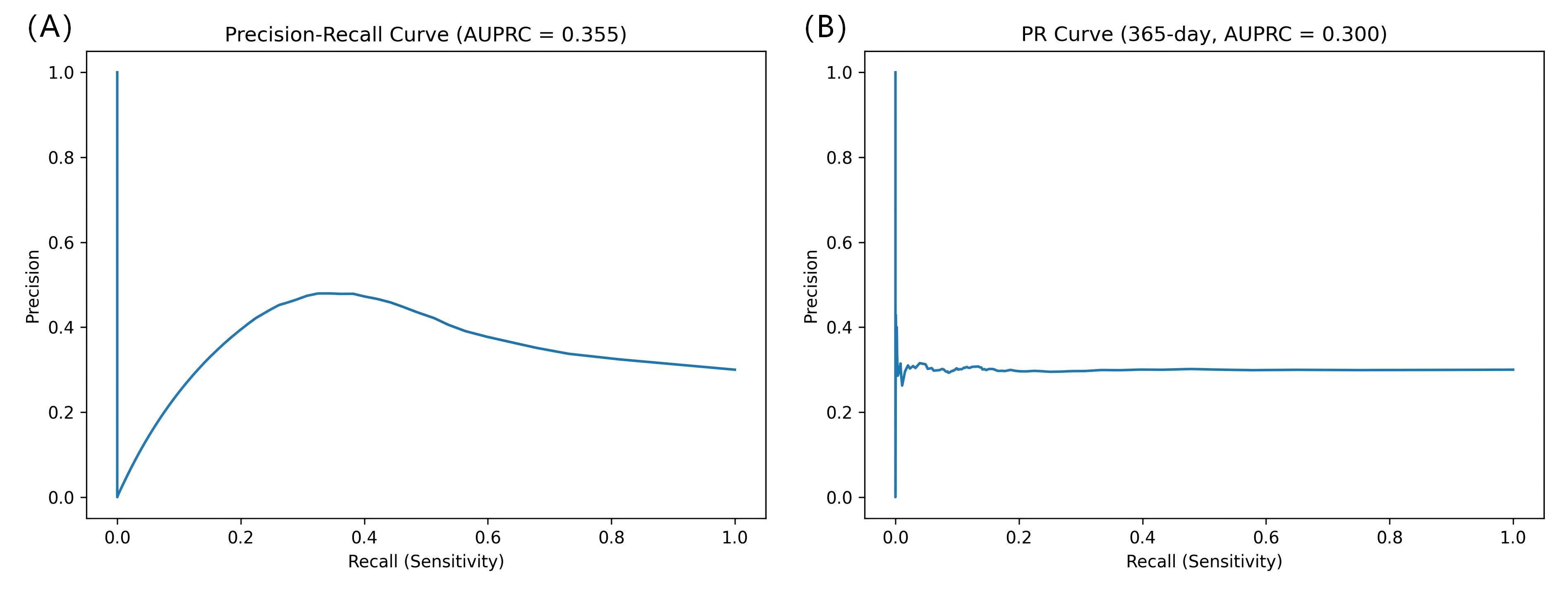

Supplement: Supplementary file 3 [file Image_2.jpeg]

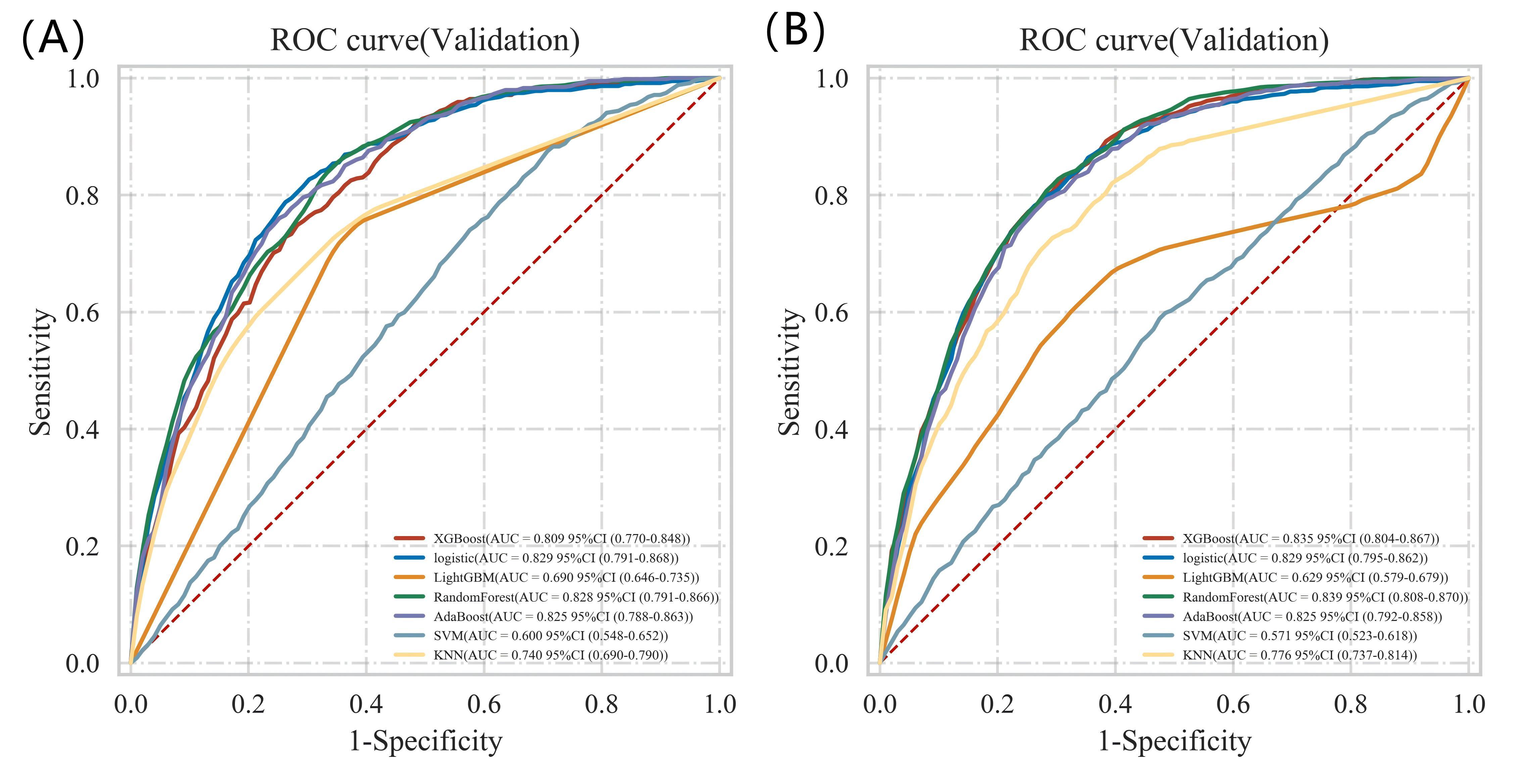

Supplement: Supplementary file 4 [file Image_3.jpeg]

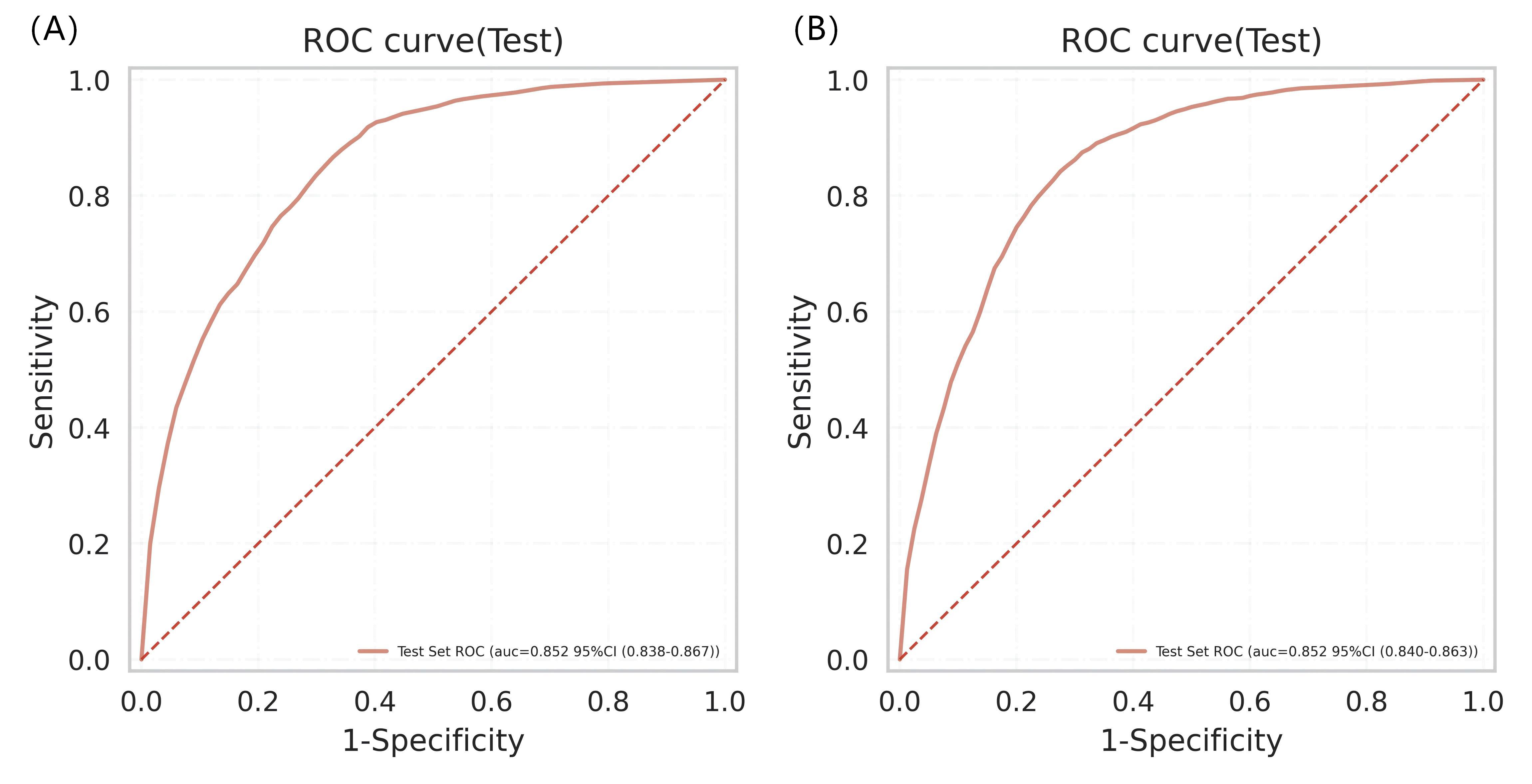

Supplement: Supplementary file 5 [file Image_4.jpeg]
